# Common scale minimal sufficient balance: An improved method for covariate‐adaptive randomization based on the Wilcoxon‐Mann‐Whitney odds ratio statistic

**DOI:** 10.1002/sim.9332

**Published:** 2022-02-17

**Authors:** Hannah Johns, Dominic Italiano, Bruce Campbell, Leonid Churilov

**Affiliations:** ^1^ Melbourne Medical School University of Melbourne Melbourne Victoria Australia; ^2^ Department of Medicine and Neurology, Melbourne Brain Centre and Royal Melbourne Hospital University of Melbourne Melbourne Victoria Australia; ^3^ Florey Institute of Neuroscience and Mental Health University of Melbourne Melbourne Victoria Australia

**Keywords:** adaptive randomization, allocation randomness, baseline covariate imbalance, clinical trial, minimal sufficient balance

## Abstract

Minimal sufficient balance (MSB) is a recently suggested method for adaptively controlling covariate imbalance in randomized controlled trials in a manner which reduces the impact on randomness of allocation over other approaches by only intervening when the imbalance is sufficiently significant. Despite its improvements, the approach is unable to consider the relative clinical importance or magnitude of imbalance in each covariate weight, and ignores any imbalance which is not statistically significant, even when these imbalances may collectively justify intervention. We propose the common scale MSB (CS‐MSB) method which addresses these limitations, and present simulation studies comparing our proposed method to MSB. We demonstrate that CS‐MSB requires less intervention than MSB to achieve the same level of covariate balance, and does not adversely impact either statistical power or Type‐I error.

## 1 INTRODUCTION

A randomized controlled trial (RCT) is considered the gold standard in medical research, and provides high quality data that can be used to reliably identify causal relationships between a clinical interventions and patient outcomes. While randomization eliminates any systematic bias in treatment trial arm assignment, the balance of clinically important prognostic variables across treatment arms can only be guaranteed “in the long run” rather than for a particular clinical trial.^1^ When such imbalance occurs, it can hamper trial interpretability, as it is not clear if an observed difference in patient outcomes is due to treatment effect or due to patients in the treatment arm being predisposed to have a better outcome.^1^, ^2^, ^3^, ^4^, ^5^, ^6^

A number of trial designs have been proposed to minimize the risks of such imbalances occurring. These include stratified and permuted block^7^ randomization, both of which allow covariate adjustment on a small number of categorical (or discretised) covariates, as well as covariate‐adjusted randomization methods,^8^ which may consider more covariates, including continuous values. A major drawback of these approaches is that they always interfere with treatment assignment by deviating from a nonbiased purely random assignment, even when covariates are perfectly balanced. As randomization is a critical method for eliminating bias in clinical trials, this unnecessary intervention is a limitation to these methods.

The minimal sufficient balance (MSB) method^9^ was developed in response to this drawback, and interferes in randomization only when covariates are sufficiently imbalanced. While MSB improves on prior randomization methods by reducing unnecessary intervention in the randomization process, it cannot consider the relative clinical importance of each covariate, nor use information about the relative magnitudes of imbalance across each covariate. MSB also ignores any imbalance information that is considered insignificant, reducing its ability to flexibly respond to covariate imbalances.

To address these limitations, we propose the novel common scale MSB (CS‐MSB) adaptive randomization procedure, which utilizes the MSB principle of only interfering with randomization process when necessary, while extending its scope and achieving better covariate balance for the same degree of interference. The remainder of this article is structured as follows. In Section 2, we provide an overview of MSB, highlight the limitations of the method, and set out a list of desirable properties that any modification of MSB should have in order to address these limitations. In Section 3, we develop CS‐MSB, addressing each of the identified desirable properties outlined in Section 2. We then present simulation studies in Section 4 to compare the performance of MSB and CS‐MSB. Finally, in Section 5 we summarize our results and outline potential directions for future research.

## 2 MINIMUM SUFFICIENT BALANCE

Minimal sufficient balance (MSB)^9^ is a recently proposed method for ensuring covariate balance in randomized controlled trials. It improves upon conventional covariate‐adjusted randomization methods by only biasing when covariate imbalance has become significant enough to warrant intervention, thus maximizing the degree of random treatment allocation. It is also capable of controlling balance on both numeric and categorical covariates without making strong assumptions about relationships between variables.

MSB operates by testing the between‐group balance of each covariate, using conventional statistical tests such as Student's *t*‐test, Pearson's $\chi^{2}$ test, or Fisher's exact test as appropriate. If a covariate is significantly imbalanced, then a vote is placed to bias randomization in whichever direction reduces the imbalance. If one treatment arm receives more votes than the other, then the probability of being assigned to that arm is set at ξ∈(0.5,1]. Otherwise, the probability of being assigned to each arm is unbiased, with probability 0.5 for each arm.

Simulation studies of MSB^10^ have demonstrated that the method successfully controls covariate imbalance when the number of covariates is large, and does not adversely impact Type‐I error. MSB been used to control covariate balance in stroke,^11^, ^12^, ^13^ Parkinson's Disease^14^ and postsurgical physiotherapy.^15^ Despite the improvement that the method offers over previously developed methods of ensuring covariate balance, it has several limitations.

### 2.1 Limitations of MSB

Because MSB intervenes based on a simple majority of votes across covariates, it does not consider the magnitude of imbalance in each covariate. This means that, should two covariates be significantly but slightly imbalanced with votes toward arm A, and one covariate be both significantly and substantially imbalanced with a vote toward arm B, then MSB will bias in favor of arm A. This also means that in the event multiple covariates are significantly imbalanced, but the votes are tied, MSB cannot break the tie and therefore does not intervene, even if one arm is substantially more imbalanced than the other.

Likewise, a simple majority vote ignores all imbalance in nonsignificant variables. This means that, should all covariates be imbalanced in favor of treatment A, but all are on the cusp of significance, then MSB will not intervene even if collectively, the consistent and nearly‐significant direction of imbalance is a strong argument for intervening. In this sense, MSB follows a strict Neyman‐Pearson interpretation of each individual covariate *P*‐value.

MSB is also limited in its ability to weight individual covariates based on importance. As it operates under simple majority voting based on individual *P*‐values, it is impossible to weight the relative importance of each covariate without directly hard‐coding the weight of each vote. Such a procedure would require a majority of less important covariates to be significantly biased in the same direction in order to overrule a single important covatiate, even if that covariate is significantly, but not substantially imbalanced. This would likely induce instability and inconsistency in the behavior of MSB, and is therefore not advisable.

Additionally, by operating at the level of statistical tests for each individual covariate, it may be difficult to provide clinical interpetation regarding how imbalanced the trial actually is in a clinical sense. While this does not inhibit the operation of MSB, it limits the ability of trialists to monitor the method during trial operation in order to ensure that it operates as expected. For example, Pearson's $\chi^{2}$ test provides a *P*‐value which is commonly used in medical research, but clinicians are not generally taught how to interpret its test statistic, making it challenging to convey the severity of the trial's imbalance at any given point. This may lead to challenges in monitoring trial imbalance in practice. Furthermore, if there are subtle differences in statistical power and other operating characteristics between each test, then on average, some covariates may be more likely to vote to intervene than others. This would, without the knowledge of trialists, add an unspecified on‐average “weight” on covariate importance. Such a situation would exacerbate MSB's inability to provide covariate weights, as there would be no way to correct such a problem should it arise.

### 2.2 Desirable properties for addressing limitations of MSB

While MSB is an important improvement in covariate‐adjustment methods for clinical trials, there are several limitations to the approach as discussed above. In order to address these limitations, we set out the following *desirable properties* for improvements to the method:

*Desirable Property* 1: The method should judge covariates on a clinically interpretable common scale compatible with all common types of univariate data. By considering each covariate on a common scale, imbalance would be measured consistently, minimizing the risk of subtle differences in statistical properties driving unexpected behavior. If this measure were to be clinically interpretable, it would also improve transparency in the method.

*Desirable Property* 2: This clinically interpretable common scale should have known, easily manipulable distributional properties. If the distributional properties of the common imbalance measure were well understood and easily transformed or otherwise mathematically manipulated, it would facilitate improvements to the MSB method that utilize these properties.

*Desirable Property* 3: The method should flexibly use covariate imbalance information to control randomization in a variety of (user‐specified) ways.

This desirable property is dependent on Properties 1 and 2 above. While MSB's *majority voting* method is a substantial improvement on previous covariate‐imbalance control methods, a common measure of imbalance would allow for weights to be attached to each covariate's vote proportional to the severity of this imbalance. Such a *weighted voting* mechanism would prioritize imbalance correction in covariates which are severely imbalanced over covariates with small imbalances. It would also allow for user‐specified covariate weights to be used in randomization, allowing for certain covariates to be considered more important than others in terms of covariate balance.

If the measure of imbalance has known and manipulable distributional properties, then instead of relying on either form of voting mechanism, MSB could consider a *pooled imbalance* approach to determine whether to intervene in the randomization process or not. This would allow the method to consider the imbalance in all covariates, rather than just those which are significantly imbalanced in its decision‐making.

Likewise, a common scale would provide greater flexibility in the nature of randomization intervention. In addition to using a *statically biased coin* to allocate patients to a favored treatment group based on a constant probability ξ∈(0.5,1], it would be possible to make the assignment probability a function of the magnitude of imbalance. This would result in a *dynamically biased coin*. For example, ξ could be proportional to the observed imbalance (with stronger coin bias when there is severe imbalance), or a step function could be used to require that imbalance be both significant and substantial before allocation probability becomes biased.

**FIGURE 1:**
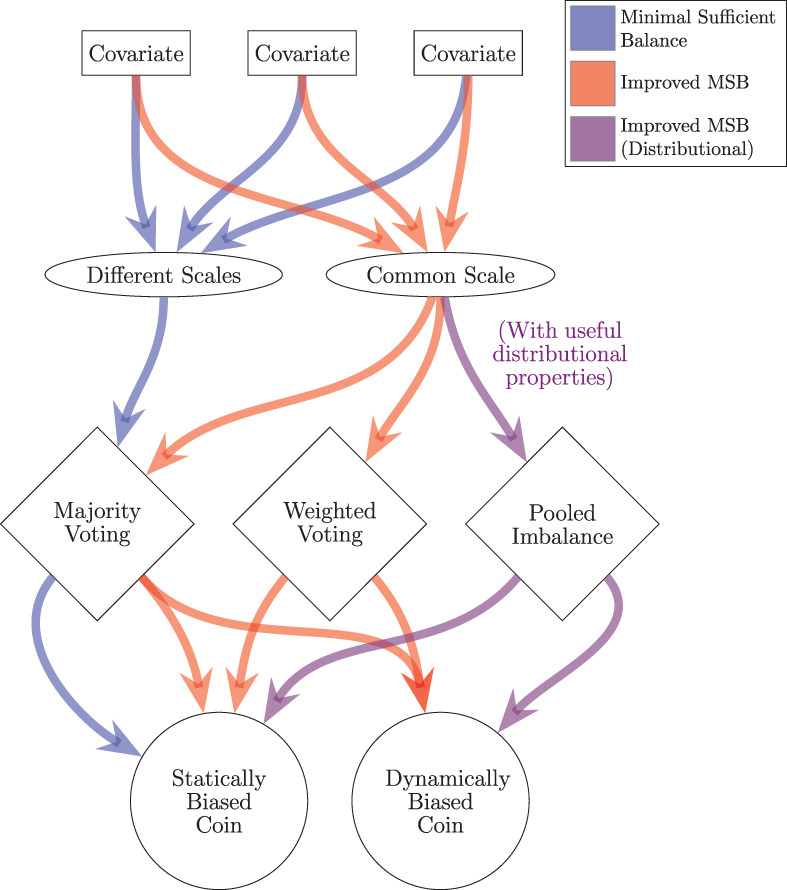
A family of minimal sufficient balance methods

Taken together, these modifications result in a family of Minimal Sufficient Balance randomization procedures, summarized in Figure 1. Depending on the application and future research, some, or none of these potential modifications to MSB may be desirable. It is therefore important that an improved version of MSB be capable of accommodating any randomization process within this family, including MSB as it was originally proposed.

*Desirable Property* 4: The method should reduce covariate imbalance at least as efficiently as MSB.

MSB uses a thresholding level of significance α to determine if covariates are sufficiently imbalanced to warrant intervention. Consequently, MSB provides a trade‐off between covariate imbalance and rate of intervention, controlled by different settings of α. By selecting this threshold, a trialist reduces a certain level of covariate imbalance at the cost of a certain rate of biased randomizations. Any improvements to MSB must be as *efficient* as MSB is, meaning that it must achieve at least the same level of covariate balance for the same degree of intervention in an otherwise purely random allocation process.

*Desirable Property* 5: The method should not adversely interfere with Type‐I error or statistical power any more than MSB.

The controlling of Type‐I error and statistical power is a cornerstone of trial design. Therefore, any improvement to MSB should not result in any further inflation of Type‐I error than occurs under MSB. Likewise, any improvement to MSB should not result in worse statistical power than under MSB.

## 3 COMMON SCALE MSB

We propose a novel extension of MSB which addresses these limitations which we name common scale MSB (CS‐MSB). While we implement our method using Wilcoxon‐Mann‐Whitney odds (WMW odds), any statistical method with known and manipulable distributional properties that is capable of handling all adjustment covariates on a common scale may be used instead. The remainder of this section is structured as follows: First, we provide an overview of the WMW odds statistic and describe the method. We then describe how CS‐MSB operates using WMW odds, using a *pooled imbalance* approach and a *statically biased coin*. Finally, we outline how similar variations on CS‐MSB may be developed to cover the whole family of Minimal Sufficient Balance methods described above.

### 3.1 The WMW odds statistic

Wilcoxon‐Mann‐Whitney odds^16^ is a modification of Agresti's generalized odds ratio (GenOR)^17^ to include information about tied values. Both WMW odds and GenOR are nonparametric test statistics which calculate the odds that, in a randomly selected pair of patients, the treatment patient has a higher score on a particular variable than the control patient. The WMW odds statistic breaks tied pairs of values by splitting them evenly between the two treatment groups. GenOR and WMW odds are closely related to the win ratio^18^ and win odds^19^ statistics respectively, both of which are topics of current attention within medical statistical literature.^20^, ^21^, ^22^, ^23^, ^24^, ^25^ WMW odds has seen recent applications in stroke research.^26^, ^27^, ^28^, ^29^, ^30^

WMW odds is capable of handling ordinal and continuous variables, and with some elementary data preprocessing is capable of handling nominal variables. It is therefore capable of handling all common types of covariate data on a common scale. It is log‐normally distributed, and therefore meets our requirement of having well‐understood and manipulable distributional properties. This property allows WMW odds to be used as a basis for considering a measure of *pooled imbalance* in CS‐MSB.

### 3.2 Common scale MSB using generalized odds ratios

The CS‐MSB randomization procedure operates as follows. Let WMWOR denote the WMW odds statistic. First, for each covariate *i*, calculate $\;|\;log(\text{WMW}\;OR_{i})\;|\;$ and $SE(log(\text{WMW}\;OR_{i}))$, testing the imbalance of the covariate across treatment groups. For continuous, ordinal or binary covariates (including two‐category categorical covariates), WMWOR can be calculated directly. To calculate WMWOR for nominal covariates, we use dummy coding for individual categories and test imbalance on the relevant categorical dummy variable. This has the added advantage of ignoring covariate imbalance when the prospective randomization cannot be improved by the new patient (eg, the covariate is imbalanced on two nominal values, but the new patient has a completely different and nominal value and thus cannot render this imbalance nonsignificant).

Second, for each covariate *i*, calculate the prospective imbalances $\;|\;log(\text{WMW}\;OR_{i}^{\left(A\right)})\;|\;$ and $\;|\;log(\text{WMW}\;OR_{i}^{\left(B\right)})\;|\;$ if the new patient were to be assigned to arm A or B respectively. Each covariate's vote is determined based on the arm which best reduces the prospective imbalance on that covariate. Under CS‐MSB, covariates place a vote on the direction of bias regardless of their individual level of significance. We next calculate the weighted averaged bias direction BD using the following formula:

(1) $$BD=\frac{1}{\sum_{i}\left[\frac{w_{i}}{SE{\left(log(\text{WMW}\;OR_{i})\right)}^{2}}\right]}\underset{i}{\sum }\left[w_{i}\;\text{sgn}\left(\left|\;log(\text{WMW}\;OR_{i}^{\left(A\right)})\;|\;-\;|\;log(\text{WMW}\;OR_{i}^{\left(B\right)})\;\right|\right)\frac{\;|\;log(\text{WMW}\;OR_{i})\;|\;}{SE{\left(log(\text{WMW}\;OR_{i})\right)}^{2}}\right],$$

where sgn(·) is the sign function, and $w_{i}$ is an optional user‐specified covariate importance weight. Likewise, we calculate the standard error of BD as

(2) $$SE{\left(BD\right)}^{2}=\frac{\sum_{i}\left[\frac{w_{i}^{2}}{SE{\left(log(\text{WMW}\;OR_{i})\right)}^{2}}\right]}{{\left[\sum_{i}\frac{w_{i}}{SE{\left(log(\text{WMW}\;OR_{i})\right)}^{2}}\right]}^{2}}.$$

If all covariates are treated with equal importance (ie, $\forall i,w_{i}=1$), this is mechanically equivalent to performing inverse‐variance weighting, and the standard error for BD can be simplified to

(3) $$SE{\left(BD\right)}^{2}=\frac{1}{\sum_{i}\left[SE{\left(log(\text{WMW}\;OR_{i})\right)}^{-2}\right]}.$$

We then determine if on average, the covariates are sufficiently imbalanced by testing if the following statement is true:

(4) $$\Phi \left(\left|\frac{BD}{SE(BD)}\right|\right)>1-\alpha /2,$$

where Φ(·) is the CDF of the standard normal distribution and α is a prespecified *P*‐value threshold for CS‐MSB intervention. If the covariates are sufficiently imbalanced, we bias randomization in favor of group A if BD<0 and in favor of group B if BD>0. The probability of assignment to the favored group is prespecified at ξ∈(0.5,1], as is the case in MSB. Likwewise, if the covariates are not sufficiently imbalanced to warrant intervention, then the arm assignment probability is set to 0.5.

By determining the contribution of each covariate's imbalance on the intervention decision based on: a combination of the size of the imbalance; the precision of the imbalance's estimate; and an optional weighting based on clinical importance, our method addresses the limitations of MSB discussed above: Our approach considers the magnitude of the imbalance in making its decision, preventing situations where two slight imbalances overrule a substantial imbalance. It also allows for explicit control over the weight placed on covariates based on clinical importance. By using a consistent test for all covariates, our method minimizes the risk that differences in statistical power could result in unexpected, uncontrolled and undesired weights being placed on each covariate. By delaying the calculation of *P*‐values until the end of the process, our method is able to consider the imbalance across all covariates, rather than discarding a combination of nonsignificant imbalances which could together justify intervention in randomization.

### 3.3 Other variations on common‐scale MSB using WMW odds

CS‐MSB may be configured to make interventions across the entire family of MSB procedures. Example variations on CS‐MSB which cover the family of MSB procedures are outlined in Table 1. Let f(x) be a user supplied function which transforms a measure of imbalance *x* into a biased coin probability ξ∈[0.5,1]. If f(x)=0.5 then the coin is unbiased for an imbalance of *x* regardless of if the imbalance is considered statistically significant. All other symbols refer to the same properties as they are defined in Section 3.2.

**TABLE 1:** Alternative extensions to MSB using WMW odds, covering the entire family of MSB methods

	Statically biased coin	Dynamically biased coin
Majority voting	Perform MSB, using WMWOR to test imbalance in each covariate. Determine votes using prospective imbalance as described in Section 3.2.	Let *x* be the sum of $w_{j}\;|\;log(\text{WMW}\;OR_{j})\;|\;$ for all significant covariates *j* that won the vote. Let ξ=f(x).
Weighted voting	For each covariate *i*, determine the vote direction and test if $\text{WMW}\;OR_{i}$ is significant. Weight each significant vote by $w_{i}\;|\;log(\text{WMW}\;OR_{i})\;|\;$. Sum the weight of significant votes for each group and intervene in favor of the group with the most weight attached.	Let $x_{A}$ be the sum of $w_{j}\;|\;log(\text{WMW}\;OR_{j})\;|\;$ for all significant covariates *j* which voted for A. Let $x_{B}$ be the sum of $w_{j}\;|\;log(\text{WMW}\;OR_{j})\;|\;$ for all significant covariates *j* which voted for B. Let $\xi =f(\;|\;x_{A}-x_{B}\;|\;)$.
Pooled Imbalance	As described in Section 3.2	Let ξ=f(|BD|).

In addition to these variations, CS‐MSB may be modified in ways not outlined in Table 1. Given the relationship between the *pooled imbalance* approach and meta‐analytic methods which are well‐understood by clinicians, and its ability to consider all covariate imbalances rather than only significant ones, we focus on the *pooled imbalance* approach for the remainder of this article.

CS‐MSB fulfills the first three *desirable properties* outlined in Section 2.2. Because WMW odds is a common and clinically interpretable scale, it satisfies *Desirable Property* 1. Because WMW odds is log‐normally distributed, it is easily manipulable and therefore satisfies *Desirable Property 2*. Finally, as the presented method may be modified to cover the entire family of potential Minimal Sufficient Balance methods (as indicated by Figure 1 and Table 1), it satisfies *Desirable Property* 3. In order to evaluate *Desirable Properties* 4 and 5, we need to compare the performance of CS‐MSB and MSB.

## 4 SIMULATION STUDIES COMPARING MSB TO CS‐MSB

To demonstrate our method and compare it to MSB, we conducted computational experiments using the EXTEND,^31^ EXTEND‐IA^32^, EXTEND‐IA TNK^27^ (TNK‐I) and EXTEND‐IA TNK Part 2^33^ (TNK‐II) clinical trials, comparing MSB and CS‐MSB. We also use a second version of the TNK‐II dataset containing additional covariates (expanded TNK‐II). Using these five datasets, we first seek to address *Desirable Property* 4 as outlined in Section 2.2 by investigating the comparative performance of MSB and CS‐MSB in terms of guaranteeing covariate balance. We then seek to address *Desirable Property* 5 by investigating the comparative performance of MSB and CS‐MSB in terms of statistical power and Type‐I error. We also seek to explore the differences between CS‐MSB when a *statically biased coin* is used, as well as when a *dynamically biased coin* is used.

### 4.1 Data

All data sources used in this study concern the use of interventions intended to restore blood flow to the brain after an ischaemic stroke. EXTEND investigated the effect of expanding the time window for the use of thrombolytics post‐stroke. EXTEND‐IA investigated the combined effect of thrombolytics and endovascular clot retrieval after stroke compared to thrombolytics alone. EXTEND‐IA TNK compared the efficacy of tenecteplase (a newer thrombolytic) to standard practice. EXTEND‐IA TNK Part 2 examined the optimal dose of tenecteplase for improving patient outcomes after stroke.

We selected different types of clinically important covariates from each of these datasets, including numeric, ordinal, nominal, and binary data. Table 2 describes the type of each covariate and provides univariate summaries of each dataset. Together, these three datasets provide examples of all common types of covariate data. As such, they provide useful illustrations for comparing the performance of MSB and CS‐MSB in a variety of real‐world settings.

**TABLE 2:** Summary of data and covariates

EXTEND (n = 225)			EXTEND‐IA TNK Part 2 (n = 300)		
Variable	Details	Distribution	Variable	Details	Distribution
Age	Patient age. Numeric	Median 76	Age	Patient age. Numeric.	Median 74
		IQR 64‐81			IQR 65‐81.25
NIHSS	Stroke severity on clinical standard	Median 11	NIHSS	Stroke severity on clinical standard	Median 16.5
	National Institutes of Health Stroke Scale.	IQR 7‐17		National Institutes of Health Stroke Scale.	IQR 10‐21
	Numeric			Numeric.
Region	Geographic region. Binary	Aus/NZ/Finland: 178	Location	Geographic region. Binary.	Metro: 259
		Asia: 47			Rural 41
Time last known well	Measure of event to treatment time. Ordinal	4.5 to 6 hours: 23	Occlusion location	Vessel occlusion location. Binary.	Internal carotid artery/basilar: 71
		6 to 9 hours: 56			Middle cerebral artery: 229
		Woke up unwell: 146
EXTEND‐IA (n=70)			Expanded covariates for TNK Part 2		
Variable	Details	Distribution	Sex	Patient sex. Binary.	Female: 141
					Male: 159
Age	Patient age. Numeric	Median 71	Atrial fibrillation	Atrial fibrillation present. Binary.	No: 219
		IQR 63‐78			Yes: 87
Stratum	Vessel occlusion location. Nominal	Internal carotid artery 17	Hypertension	Hypertension present. Binary	No: 103
		First segment middle cerebral artery 44			Yes: 197
		Second segment middle cerebral artery 9
Occlusion Location	Stroke severity on clinical standard	Median 15	Lipid disorders	Lipid disorders present. Binary.	No: 182
	National Institutes of Health Stroke Scale.	IQR 12‐19			Yes: 118
	Numeric.
EXTEND‐IA TNK (n = 202)			Previous stroke or TIA	Patient previously had a stroke or	No: 262:
				Transient Ischaemic Attack. Binary.	Yes: 38
Variable	Details	Distribution	Ischaemic heart disease	Ischaemic heart disease present. Binary.	No: 233
					Yes: 67
Age	Patient age. Numeric.	Median 74	Diabetes mellitus	Diabetes mellitus present. Binary.	No: 241
		IQR 65‐81.25			Yes: 59
NIHSS	Stroke severity on clinical standard	Median 17	Peripheral vascular disease	Peripheral vascular disease present. Binary.	No: 289
	National Institutes of Health Stroke Scale.	IQR 12‐22			Yes: 11
	Numeric.
Occlusion location	Vessel occlusion location. Nominal	Internal carotid artery/basilar: 55
		First segment middle cerebral artery: 119
		Second segment middle cerebral artery: 28			

In each dataset, patient outcomes were reported using the modified Rankin scale (mRS),^34^ a widely used and clinically interpretable ordinal measure of functional ability ranging from 0 (no symptoms of stroke) to 6 (death). Each successive dichotomization on this scale has well‐understood clinical meaning.^35^ For example, an mRS score between 0 and 2 is interpreted as an ability to live independently, while an mRS score between 0 and 3 is interpreted as ability to walk without assistance of another person.

### 4.2 Methods

To address the aims outline above, we performed three simulation experiments. The first two of these compare CS‐MSB with MSB and address *Desirable Properties* 4 and 5 outlined in Section 2.2. They focus on CS‐MSB with a *statically biased coin*, as it is analogous to the established MSB method which also uses a statically biased coin. The third simulation experiment compares the behavior of CS‐MSB with a *statically biased coin* to CS‐MSB with a *dynamically biased coin*. In each simulation, we used the data from the individual trials described above in their original order to examine the hypothetical scenarios where the randomization was conducted according to either MSB or CS‐MSB.

#### 4.2.1 Comparing MSB with CS‐MSB using a statically biased coin

To address *Desirable Property* 4, we used a full‐factorial design to examine the effect of α and ξ on the degree of covariate imbalance and intervention rate using three individual datasets. We described the covariate imbalance at the end of the trial using both the WMW odds and effect size statistics related to the original MSB's voting methods (Cohen's *d* for continuous data; Pearson's $\chi^{2}$ statistic for categorical data), and recorded the *P*‐values associated with each covariate imbalance. For ordinal data, which was not originally discussed in the context of MSB, we used WMW odds and its associated *P*‐value to describe covariate imbalance. We also recorded the Intervention Rate (IR, defined as the proportion of randomizations where a biased coin was used) for both MSB and CS‐MSB. For each combination of dataset, ξ and α, we performed 5000 simulations.

To address *Desirable Property* 5, we used Rubin's counterfactual framework^36^ and provided each patient with counterfactual outcome probabilities conditional on: patient assignment to treatment (assuming treatment effect exists); patient assignment to control (assuming treatment effect exists); and no treatment effect (ie, the null hypothesis is true). Each of these sets of outcome probabilities were generated using distance‐weighted *k*‐nearest neighbors,^37^ trained using only patients who were assigned to control (for probability of outcome given assignment to control), then only patients who were assigned to treatment (for probability of outcome given assignment to treatment), and finally all patients (assuming no treatment effect).

For computational feasibility, we chose values of α to achieve a target intervention rate of 5%, 15%, and 25% based on the results of the simulations addressing *Desirable Property* 4, selecting the value of α which resulted in a median intervention rate closest to each target (representing a usual case), a 75th percentile intervention rate closest to each target (representing setting the intervention rate in a conservative manner), and a 25th percentile intervention rate closest to each target (representing setting the intervention rate in an anticonservative manner). For each combination of target intervention rate and quantile, we ran 5000 simulations. At the end of each simulated trial, we tested for a significant treatment effect using ordinal logistic regression, and logistic regression at each dichotomous cutpoint. We recorded the proportion of significant outcomes (ie, the empirical power/Type‐I error) for each test.

#### 4.2.2 Comparing the behavior of CS‐MSB using statically and dynamically biased coins

To illustrate the behavior of a dynamically biased coin, we followed the same simulation process described above to evaluate *Desirable Property 4*. We used a *linearly biased coin* that was directly proportional to the strength of the pooled bias, with biased coin probability ξ given by the function

(5) $$f(x)=\min\left\{0.5+sx,\;1\right\},$$

where x=|BD| and *s* is a positive number giving the strength of the proportionality. In a similar manner to the statically biased coin case, we performed full‐factorial design over α and *s*, where s=1,4,7,…,13,16. As the probability associated with a biased coin may change from one random assignment to the next, we require a measure for the strength of intervention which incorporates not only the rate of intervention, but also the magnitude. We therefore introduce *Expected Bias* (EB) as such a measure, defined as the expected deviation of a randomly selected patient's randomization probability from 0.5, and is given by

(6) $$EB=\frac{1}{N}\overset{N}{\underset{i=1}{\sum }}\left|\text{Prob}(X_{i}=A)-0.5\right|,$$

where $X_{i}$ is the arm assigned to observation *i* and *A* is one of the treatment arms. For a statically biased coin with bias strength ξ, this value may be derived from the intervention rate IR and is given by

(7) EB=IR×(ξ−0.5).

### 4.3 Results: Comparing MSB with CS‐MSB

**FIGURE 2:**
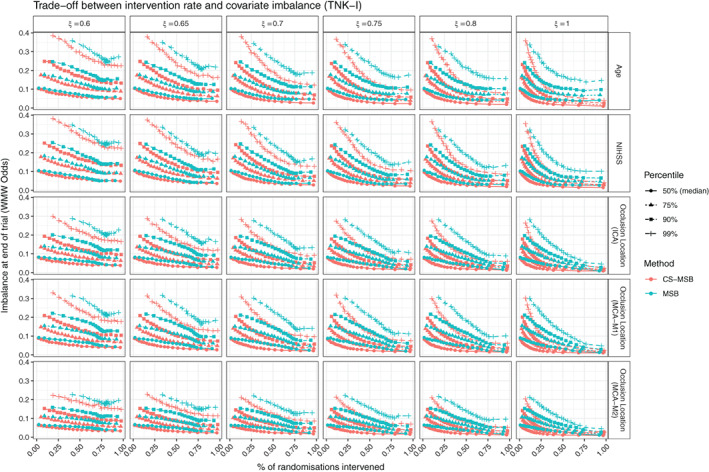
Trade‐off curve between covariate imbalance in the TNK dataset. Imbalance is measured using WMW odds. Curves shown give worst‐case estimates for this trade‐off (ie, highest percentile imbalance for highest percentile intervention rate. An ideal trial would appear in the lower left‐hand corner, with no imbalance and no intervention.)

**FIGURE 3:**
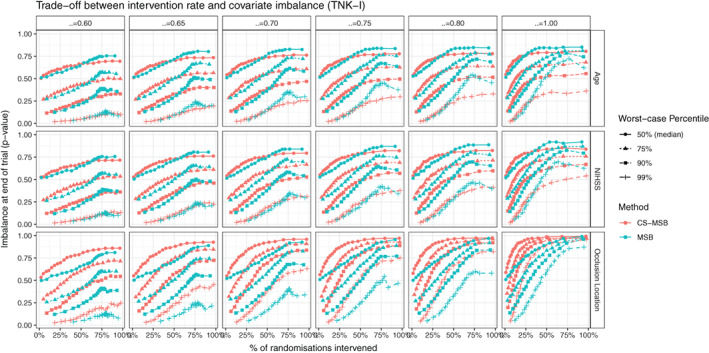
Trade‐off curve between covariate imbalance in the TNK dataset. Imbalance is measured using conventional statistical tests and reported using *P*‐values. Curves shown give worst‐case estimates for this trade‐off (ie, lowest percentile *P*‐value for highest percentile intervention rate. An ideal trial would appear in the upper left‐hand corner, with no statistically significant imbalance at any significance threshold and no intervention.)

*Desirable Property* 4 requires that any improvement to MSB be at least as efficient as MSB. CS‐MSB was more efficient at controlling covariate imbalance, with less imbalance at the end of the trial for the same rate of intervention. The magnitude of imbalance at the end of the trial (measured using the *WMW* odds as set out in Section 3.2, shown in Figure 2. Equivalent figures for all other datasets are provided in the supplementary material) was consistently lower for CS‐MSB. When measured using conventional univariate statistical tests CS‐MSB resulted in consistently higher *P*‐values than MSB (meaning less significant imbalance) when the intervention rate was low (Figure 3. Equivalent figures for all other datasets are provided in the supplementary material). For high intervention rates, the NIHSS covariate in the TNK dataset, and the Age covariate in all datasets saw a reversal in these results. The precise threshold at which this reversal occurred varied with the dataset and the value of ξ. The required intervention rate for MSB to outperform CS‐MSB in the Age covariate was approximately 60% for the EXTEND and TNK‐II datasets, and anywhere from 25% to 50% depending on ξ in the TNK and EXTEND‐IA datasets, with low values of ξ requiring a higher intervention rate. The required intervention rate for MSB to outperform CS‐MSB on the NIHSS covariate in TNK‐I was approximately 35%. An intervention rate in excess of 90% was required to see this behavior in the expanded TNK‐II dataset. Each of these thresholds represents an extremely high level of intervention, indicating that MSB only outperformed CS‐MSB when the imbalance sensitivity α is so high that the primary purpose of MSB (reduce the degree of intervention in an otherwise purely random allocation process) is not being achieved.

For MSB, we also observed a nonmonotonic relationship between intervention rate and imbalance at the end of the trial, with high intervention rates (approximately 75% or more ) resulting in a decrease in performance. There are several possible explanations for this behavior. First, this behavior could be driven by the relationship between α and intervention rate for MSB (Figure 5), where extremely high values of α result in more tied votes, thus reducing the intervention rate. This would mean that extremely high intervention rates in MSB may correspond to a lower sensitivity to imbalance, explaining why imbalance appears to worsen at high intervention rates. Second, this behavior could be driven by larger variability in the MSB method (discussed in Section 4.5). Even for very low values of α, it was possible for MSB to achieve intervention rates in excess of 75% meaning that the high intervention rate for MSB contains a greater proportion of extreme instances of low‐sensitivity α. As extreme sample percentiles exhibit higher variability than less extreme percentiles, this behavior could also be an artifact of the finite number of simulations performed. This explanation is supported by the fact that the nonmonotonicity becomes more pronounced at extreme percentiles. Regardless of the cause, this observed behavior occurs at extremely high intervention rates which, in practice, defeat the purpose of performing MSB over other covariate‐adjustment methods.

Across all five tested datasets, MSB only outperforms CS‐MSB on a small number of variables and only achieves this when the intervention rate is extremely high. At practically desirable intervention rates, CS‐MSB consistently provides better covariate imbalance than MSB for the same level of intervention within a trial.

**FIGURE 4:**
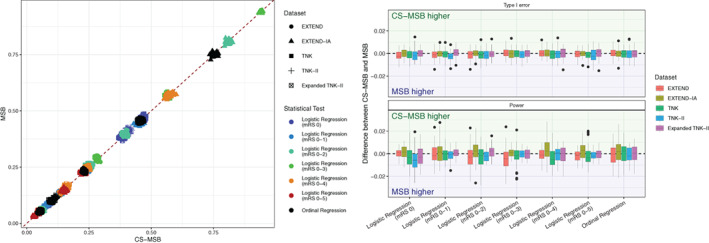
Agreement between CS‐MSB and MSB. The left panel demonstrates the agreement on power and Type‐1 error between MSB and CS‐MSB. The right panel demonstrates that any differences are small and centered around zero

*Desirable Property*5 requires that any improvement to MSB not adversely impact either statistical power or Type‐I error. Overall, there was almost perfect agreement between MSB and CS‐MSB in terms of statistical power and Type‐I error, with Lin's concordance correlation coefficient exceeding 0.995 on all outcomes. Reduced major axis regression analysis demonstrated no substantial fixed or proportional bias in the results, with all intercepts and slopes either not significantly different, or negligibly deviating from values of 0 and 1 respectively. The largest significant deviation from an intercept of 0 was −0.0011, and the largest significant deviation from a slope of 1 was a slope of 1.007. The absolute size of the disagreement between MSB and CS‐MSB was small, with most having a difference in power less than 0.01, or a difference in Type‐1 error less than 0.005. Neither method had values which were consistently higher than the other, resulting in differences which were centered around 0. (Figure 4).

In all cases the magnitude of difference in empirical power was negligible, and there was no evidence to suggest that one method had consistently higher power or Type‐I error than the other. In all five investigated datasets, CS‐MSB therefore controls Type‐I error just as well as MSB, and achieves equivalent statistical power.

### 4.4 Results: Comparing statically and dynamically biased coins

There was no definitive improvement in efficiency when comparing CS‐MSB with a statically biased coin to CS‐MSB with a linearly biased coin as given in Equation 5. In the expanded TNK‐II dataset, a linearly biased coin appeared to perform marginally better than a dynamically biased coin at extreme worst‐case percentiles, though this pattern was reversed in the EXTEND dataset. Figures showing these results are available in the supplementary material. In all cases, differences in efficiency were small, were most noticeable in estimates for extreme worst‐case scenarios, and varied in direction across datasets.

There are several potential explanations for the inconsistency in relative performance for these measures. First, it is possible that the relative performance of a linearly and statically biased coins varies with either the sample size or the number or type of covariates. It could also be the case that these results are a statistical artifact driven by the finite number of simulations performed.

### 4.5 Additional results concerning parameter selection for MSB and CS‐MSB with a statically biased coin

Upon further analysis of the results of our simulation studies, we discovered that our simulation for examining *Desirable Property* 4 also revealed an unexpected pattern in the relationship between α and the resulting intervention rate. The relationship between α and the intervention rate was nonlinear for both MSB and CS‐MSB, and depended on both ξ and the trial dataset the method was being applied to. This relationship was also qualitatively different between MSB and CS‐MSB.

**FIGURE 5:**
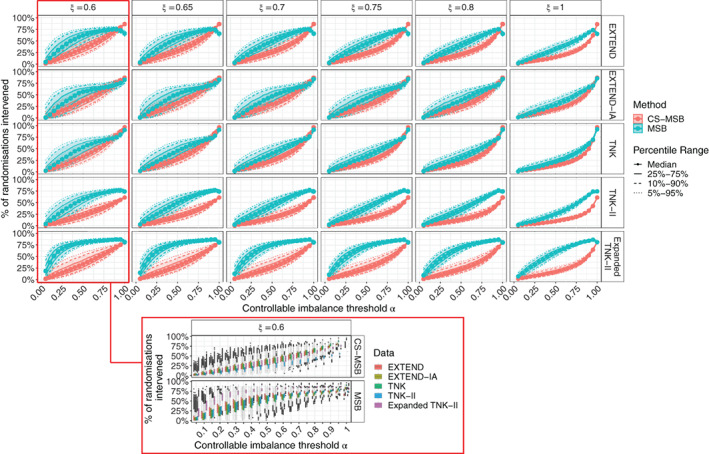
Calibration curves for MSB and CS‐MSB showing the intervention rate for both methods with various settings of ξ and α across three datasets. Bands around median intervention rate show the middle 50% (interquartile range), middle 80% and middle 90% of intervention rates for a given setting and dataset. The red bordered boxplots illustrate the differences in calibration curves across different datasets

In general, CS‐MSB required α to be set at a higher value to achieve the same intervention rate as MSB. CS‐MSB was substantially more consistent in its rate of intervention, with less variability in intervention rates for a prespecified value of α (Figure 5).

The requirement of a higher value of α in CS‐MSB is likely due to the fact that the method uses a *pooled imbalance* approach to determine if intervention should occur. CS‐MSB effectively “averages” imbalances across covariates, which naturally inclines BD to tend toward zero as a result of summing positive and negative values to indicate vote direction. This therefore requires a higher value of α to achieve the same sensitivity to imbalance. It is not immediately clear why CS‐MSB has more consistent performance than MSB.

## 5 DISCUSSION

In this article, we proposed a family of common scale minimal sufficient balance randomization procedures (CS‐MSB), systematically examining one which used a *pooled imbalance* and *statically biased coin* approach, and illustrating one with a *pooled imbalance* and *dynamically biased coin* approach. We then used simulation studies to demonstrate that CS‐MSB more efficiently reduces covariate imbalance than MSB, and does not adversely impact either statistical power or Type‐I error. We did not observe any further improvements in our illustration of a *dynamically biased coin*. We also demonstrated that CS‐MSB is more consistent in the rate at which it intervenes in a trial when compared to MSB.

There are several potential explanations for observed efficiency improvement. First, by dichotomizing nominal variables at each randomization to make them compatible with WMW odds, CS‐MSB does not intervene in significant imbalance on nominal variables when there is no chance of intervention improving randomization. This would likely lead to improved efficiency in CS‐MSB even if the method used MSB's simple voting mechanism. However, this would only explain the improvements observed in the TNK‐I trial dataset. Second, both CS‐MSB and MSB implicitly assume that covariates are not correlated, an assumption which is rarely accurate on real datasets and is not accurate in EXTEND, TNK‐I or TNK‐II. This may result in counterintuitive properties, such as where balancing one covariate may unbalance another, or conversely may rebalance several covariates. By considering all covariates at once, CS‐MSB may become more efficient in the presence of correlated covariates. Finally, the *pooled imbalance* approach allows CS‐MSB to intervene when multiple covariates agree on bias coin direction, but have not yet become significantly imbalanced. This may allow CS‐MSB to make more efficient interventions, as the method will be more likely to intervene when a single intervention would correct multiple imbalances.

Our simulation examining the comparative efficiency of MSB and CS‐MSB also demonstrated that for both randomization methods, the relationship between α and the intervention rate depends on the population used in the trial. This finding has important implications from a trial design perspective, as it suggests that setting the imbalance significance threshold α based on anticipated behaviors of univariate summary statistics may be unreliable. Instead, we recommend that the imbalance significance threshold α should be set through simulation study. In practice, this would mean setting α to achieve some target intervention rate with a certain probability. For example, setting α to achieve a particular 80th percentile intervention rate would mean accepting a 1‐in‐5 chance of intervening more often than the specified rate. This would both eliminate the risk of wrongly considering α to be an intuitive proxy for the intervention rate, and would more accurately represent the inherent stochasticity of the MSB family of randomization methods.

While we have demonstrated that CS‐MSB outperforms MSB, there are several areas where further improvements could be made. First, neither MSB or CS‐MSB provide any sort of guarantee regarding the balance of sample sizes within treatment arms, meaning that one treatment arm may be randomly allocated more patients than the other. Improvements to the method which counteract this (eg, providing a burn‐in period or combining adaptive randomization with allocation based on a random, concealed table) would be clinically important. Second, both MSB and CS‐MSB assume that all covariates are uncorrelated, which may be unrealistic in practice. Further improvements may be made by modifying either MSB or CS‐MSB to incorporate information about correlation among covariates. Third, both MSB and CS‐MSB are defined for two‐group RCT designs, and cannot be readily applied to multiarm designs. Modifications to the method to handle multiarm RCT designs would further improve the utility of the randomization procedure. Similarly, extensions which allow for unequal treatment allocation ratios across treatment arms would also increase the utility of both MSB and CS‐MSB.

Additionally, in this article, we considered CS‐MSB using a *pooled imbalance* approach. While this approach improved performance when compared to MSB, further research is needed to determine if additional improvements could be made by applying a different randomization procedure in the MSB family. Likewise, while we performed a head‐to‐head comparison between MSB and CS‐MSB, we did not compare CS‐MSB to other covariate‐adjusted randomization procedures. We also did not explore sensitivity analyses using rerandomization tests as is often required by regulatory bodies for trials which utilize adaptive randomization procedures.^1^ Further research is also needed to address both of these points.

Furthermore, the use of a dynamically biased coin is an area which requires a substantial amount of further exploration. While a linearly biased coin did not show consistent improvements over a statically biased coin, there are many possible functions which could have been applied instead where further improvement may have been observed. Likewise, the *expected bias* metric applied in this article assumes an equal weight between the intervention rate and the strength of the biased coin. Had some other metric been used that differently captured the multifaceted nature of a dynamically biased coin, the results presented in Section 4.3 may have been different. Both of these present opportunities for future research.

In summary, in this article, we developed an improvement to the minimal sufficient balance randomization, which requires less intervention in a trial to maintain the same level of covariate imbalance. Our improved method is also more consistent than MSB, and does not adversely impact either power or Type‐I error.

## Supporting information

Supporting Information

## Data Availability

The underlying clinical data used in this study are not available due to ethical and legal requirements. The simulation data supporting the findings of this study are available from the corresponding author upon reasonable request.
