# Supplementary material for: Common scale minimal sufficient balance: An improved method for covariate‐adaptive randomization based on the Wilcoxon‐Mann‐Whitney odds ratio statistic
Source: Stat Med. 2022 Feb 17;41(10):1846–61. doi: 10.1002/sim.9332 (PMC9303921; doi:10.1002/sim.9332)
Supplement: Supplementary file 1 — Supporting Information [file SIM-41-1846-s001.pdf]

1 Supplementary material for Section 4.3

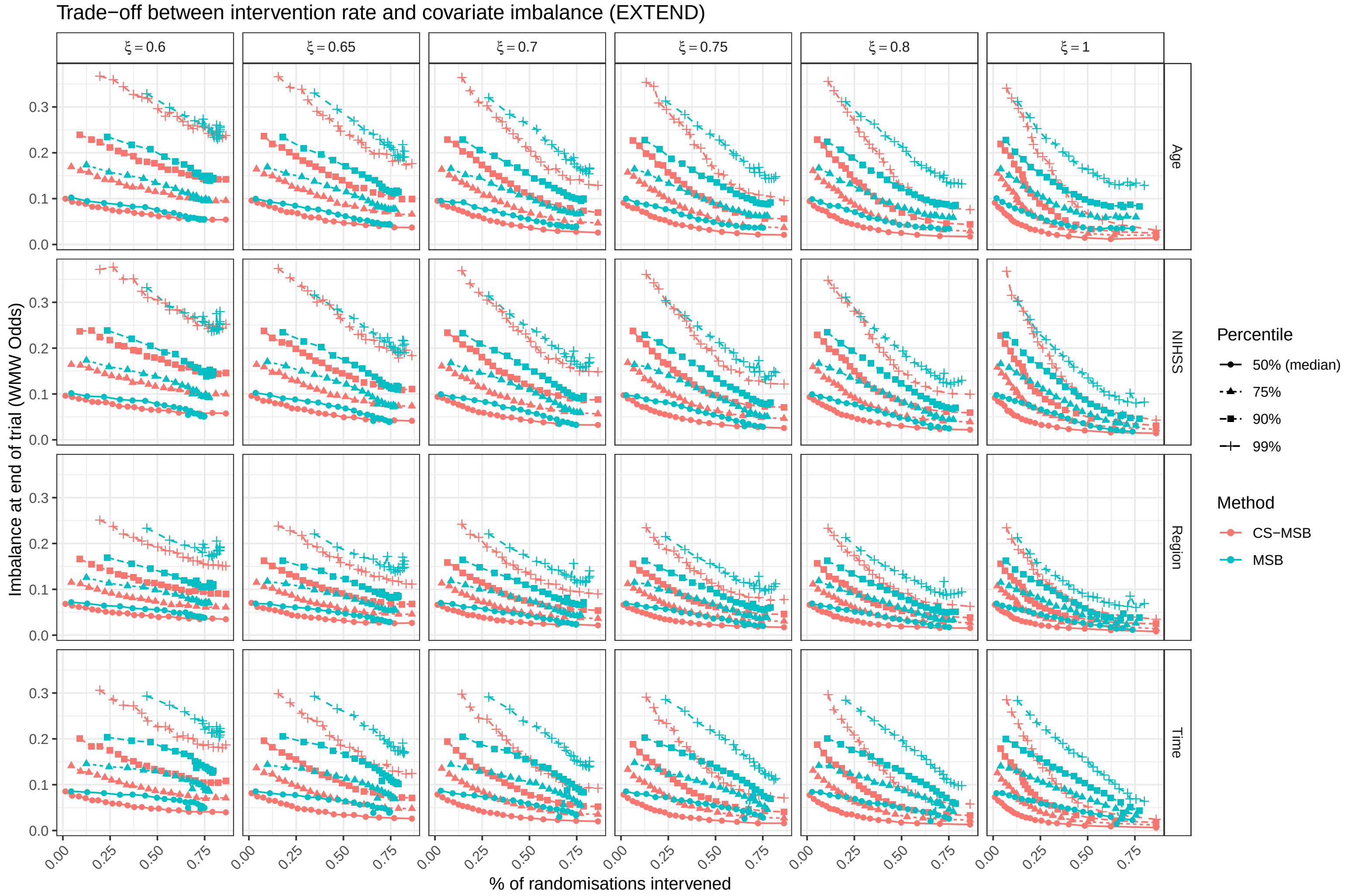

Figure 1: Trade-off curve between covariate imbalance in the EXTEND dataset. Imbalance is measured using WMW Odds. Curves shown give worst-case estimates for this trade-off (i.e. highest percentile imbalance for highest percentile intervention rate. An ideal trial would appear in the lower left-hand corner, with no imbalance and no intervention.)

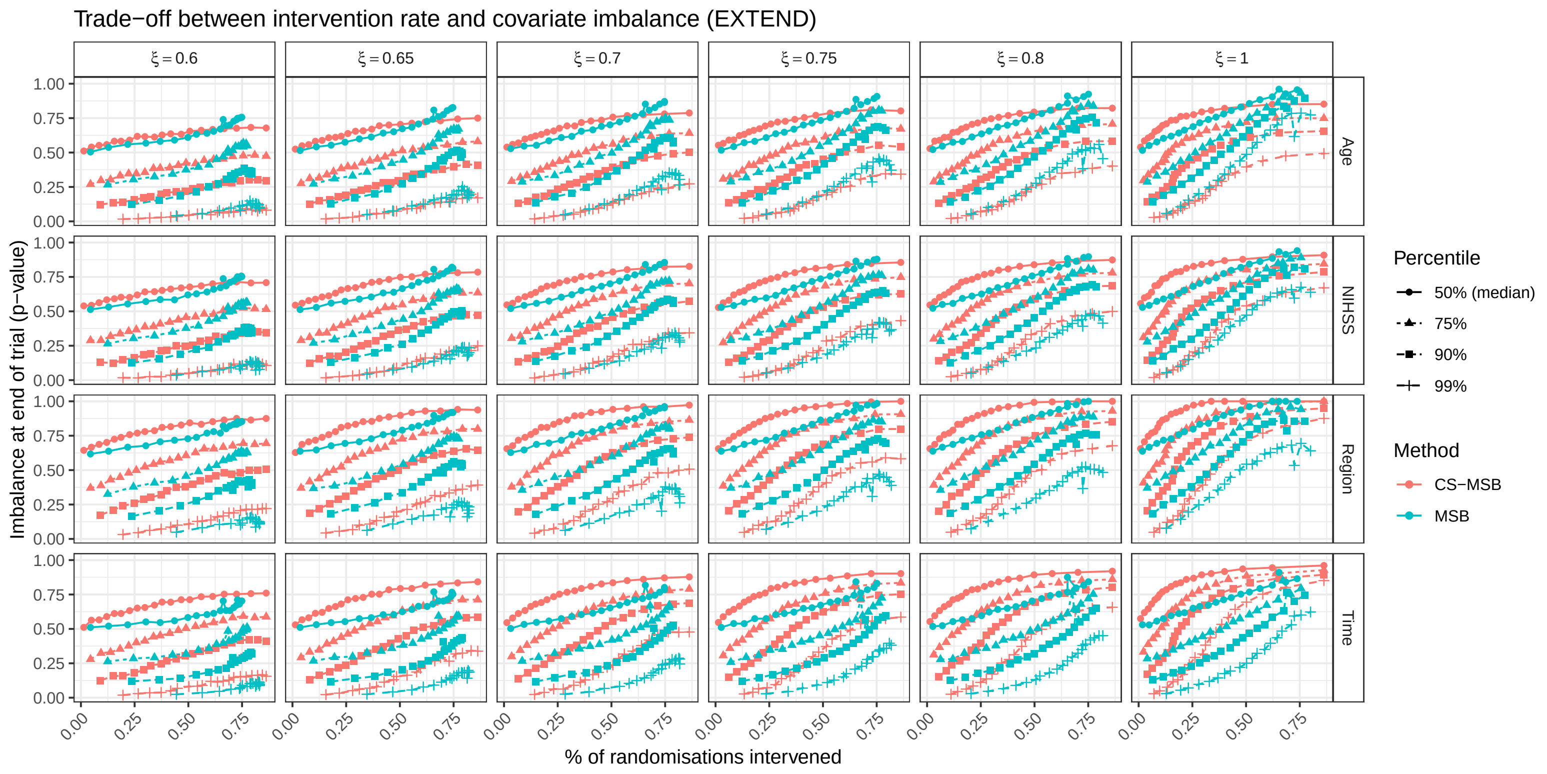

Figure 2: Trade-off curve between covariate imbalance in the EXTEND dataset. Imbalance is measured using conventional statistical tests and reported using p-values. Curves shown give worst-case estimates for this trade-off (i.e. lowest percentile p-value for highest percentile intervention rate. An ideal trial would appear in the upper left-hand corner, with no statistically significant imbalance at any significance threshold and no intervention.)

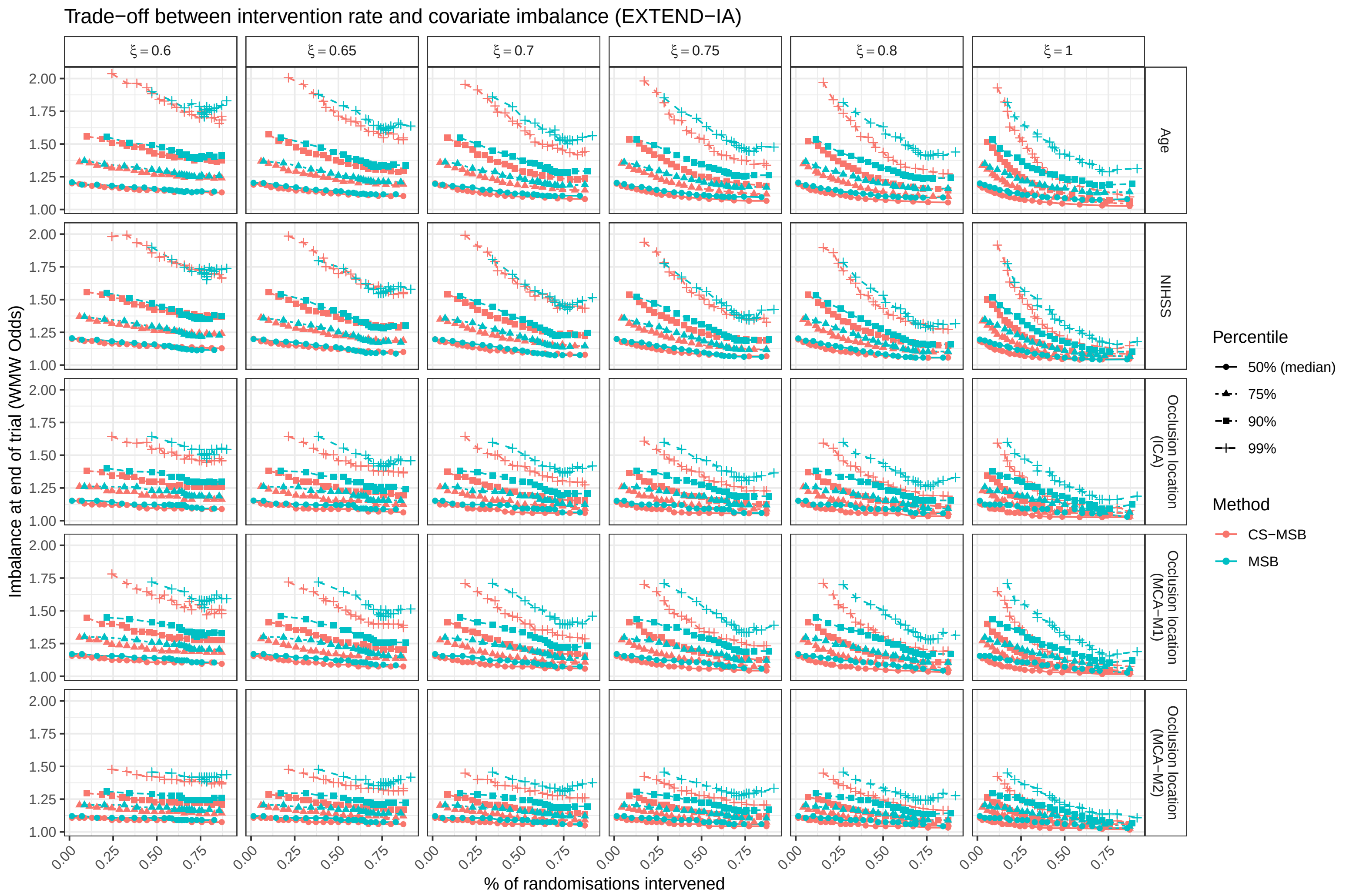

Figure 3: Trade-off curve between covariate imbalance in the EXTEND-IA dataset. Imbalance is measured using WMW Odds. Curves shown give worst-case estimates for this trade-off (i.e. highest percentile imbalance for highest percentile intervention rate. An ideal trial would appear in the lower left-hand corner, with no imbalance and no intervention.)

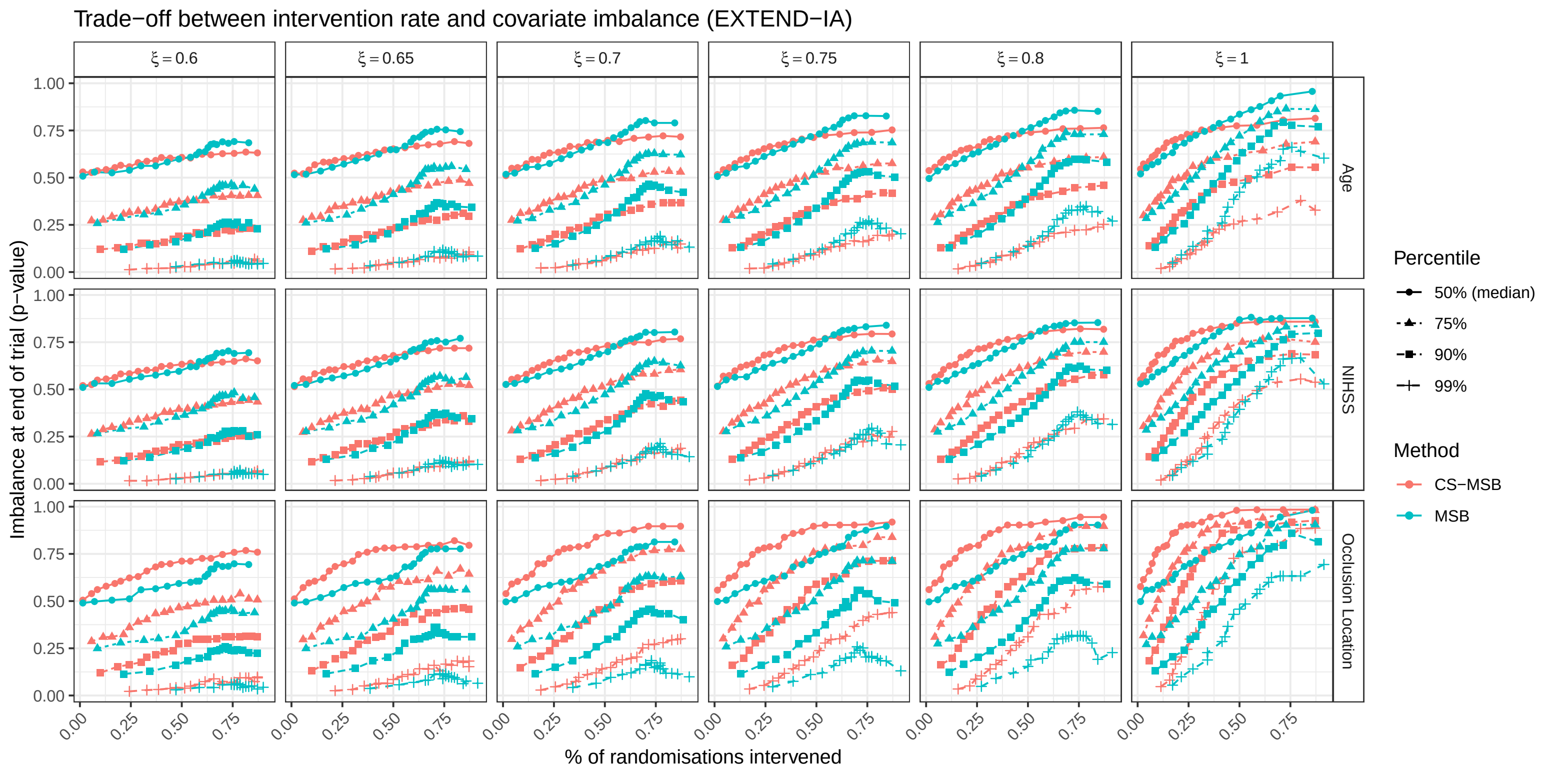

Figure 4: Trade-off curve between covariate imbalance in the EXTEND-IA dataset. Imbalance is measured using conventional statistical tests and reported using p-values. Curves shown give worst-case estimates for this trade-off (i.e. lowest percentile p-value for highest percentile intervention rate. An ideal trial would appear in the upper left-hand corner, with no statistically significant imbalance at any significance threshold and no intervention.)

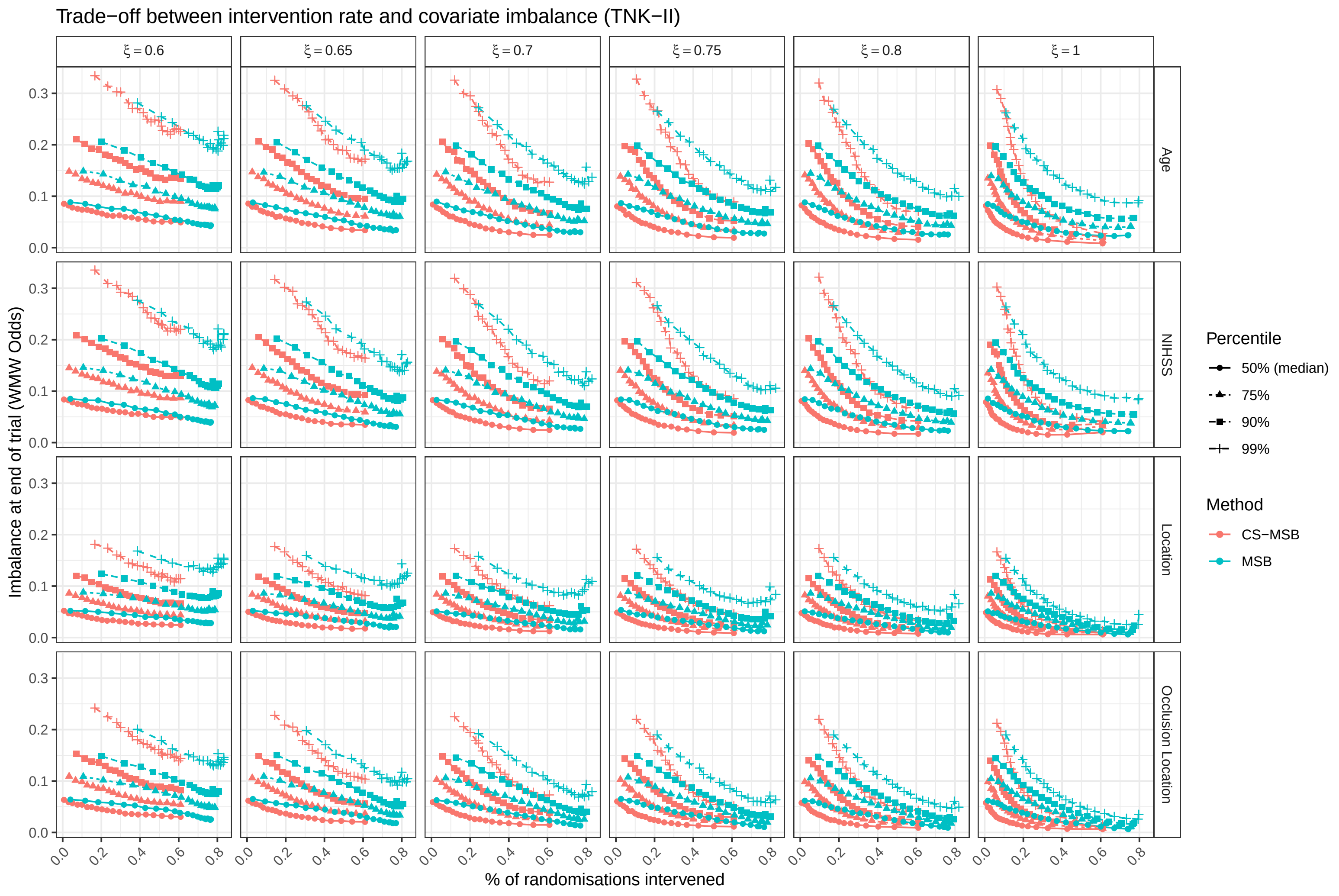

Figure 5: Trade-off curve between covariate imbalance in the TNK Part 2 dataset. Imbalance is measured using WMW Odds. Curves shown give worst-case estimates for this trade-off (i.e. highest percentile imbalance for highest percentile intervention rate. An ideal trial would appear in the lower left-hand corner, with no imbalance and no intervention.)

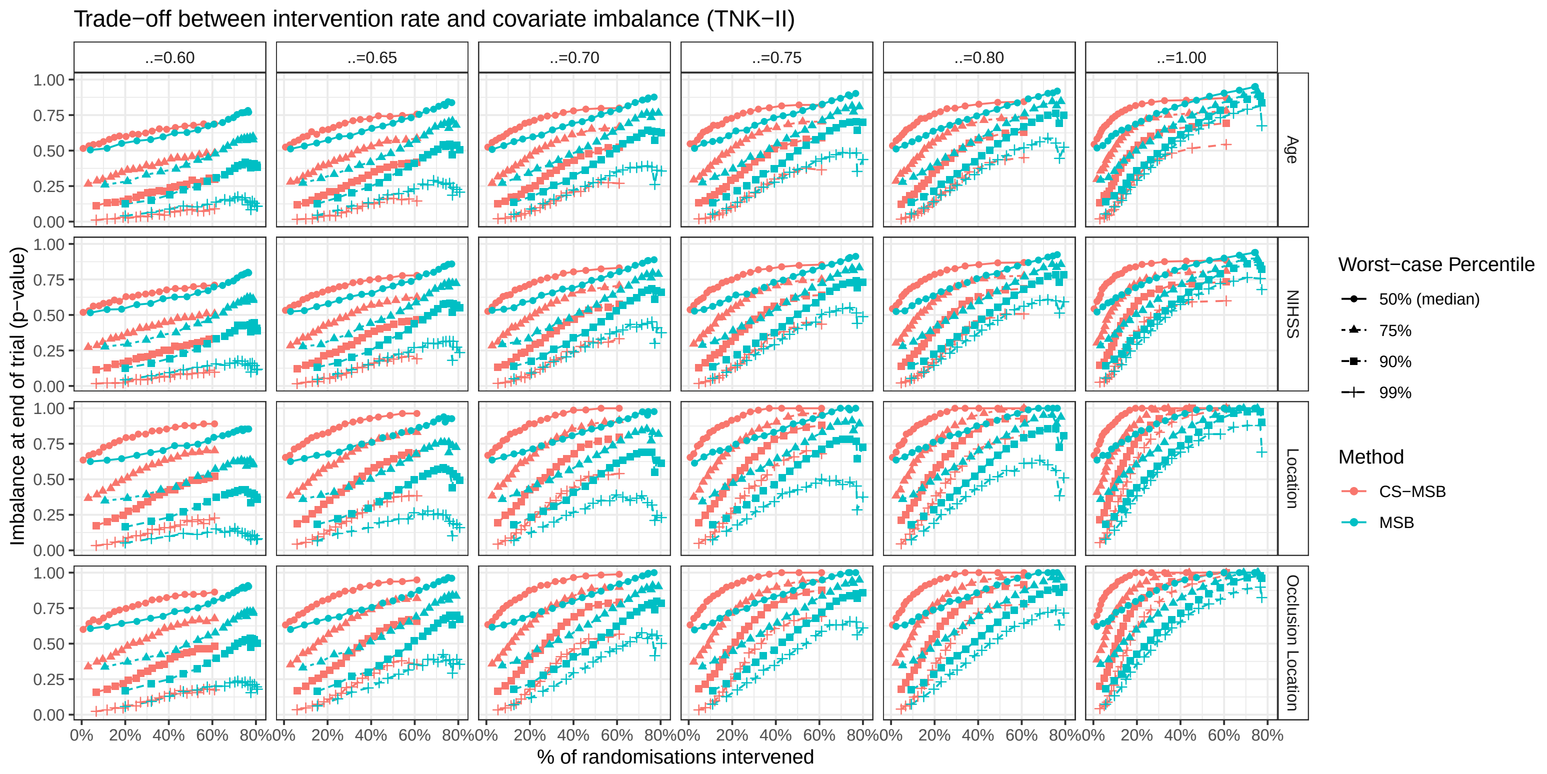

Figure 6: Trade-off curve between covariate imbalance in the TNK Part 2 dataset. Imbalance is measured using conventional statistical tests and reported using p-values. Curves shown give worst-case estimates for this trade-off (i.e. lowest percentile p-value for highest percentile intervention rate. An ideal trial would appear in the upper left-hand corner, with no statistically significant imbalance at any significance threshold and no intervention.)

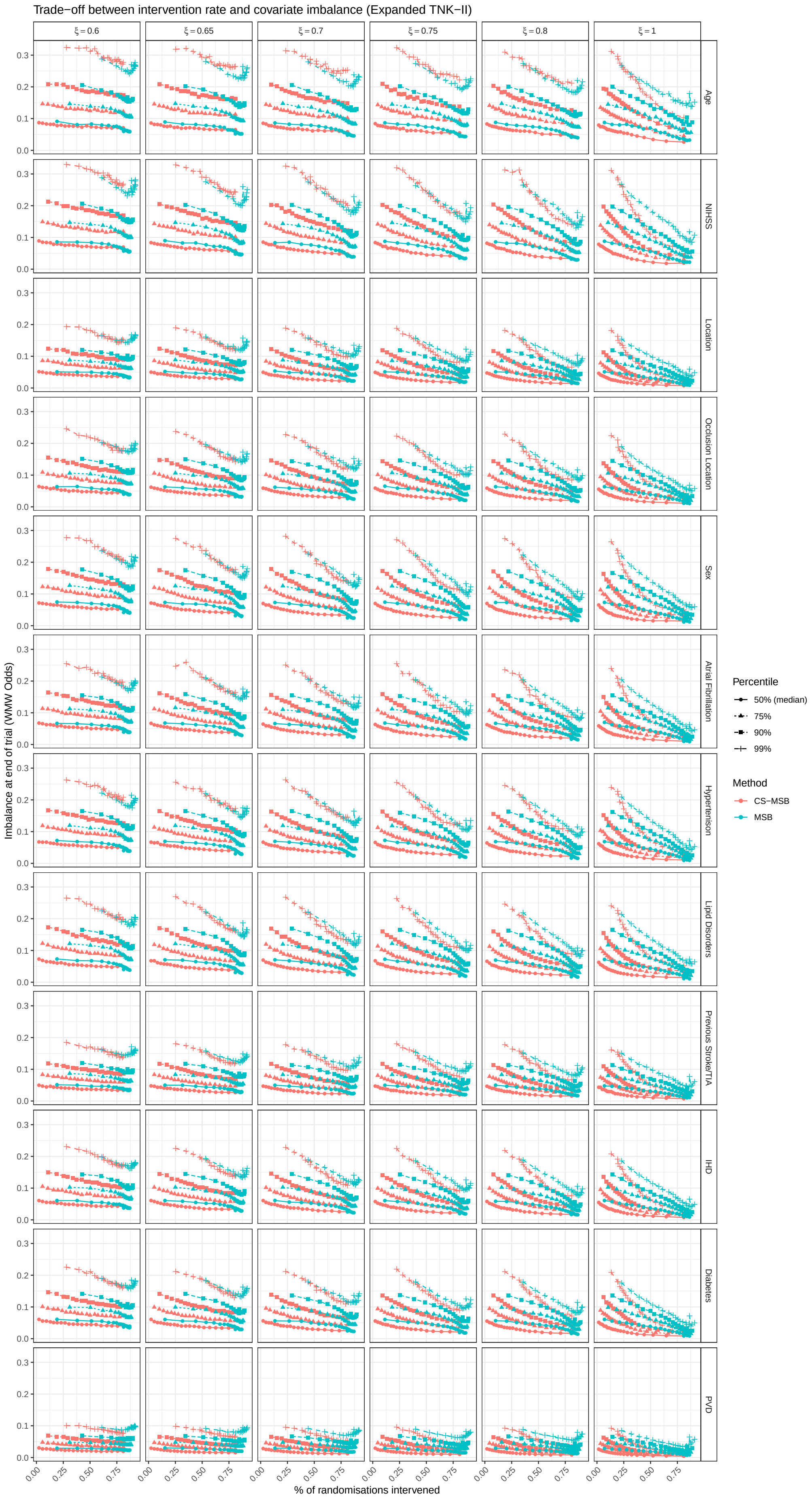

Figure 7: Trade-off curve between covariate imbalance in the expanded TNK Part 2 dataset. Imbalance is measured using WMW Odds. Curves shown give worst-case estimates for this trade-off (i.e. highest percentile imbalance for highest percentile intervention rate. An ideal trial would appear in the lower left-hand corner, with no imbalance and no intervention.)

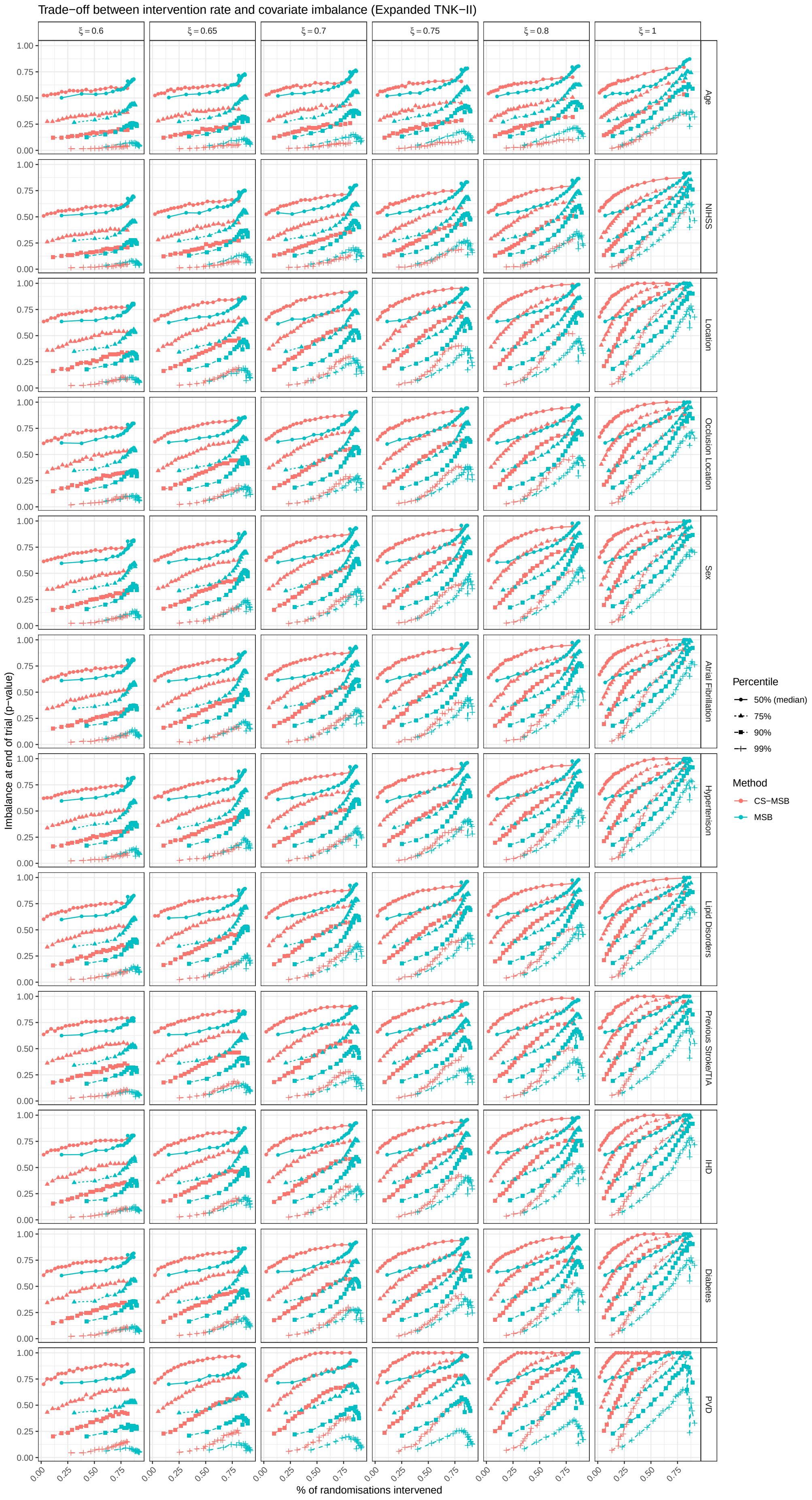

Figure 8: Trade-off curve between covariate imbalance in the expanded TNK Part 2 dataset. Imbalance is measured using conventional statistical tests and reported using p-values. Curves shown give worst-case estimates for this trade-off (i.e. lowest percentile p-value for highest percentile intervention rate. An ideal trial would appear in the upper left-hand corner, with no statistically significant imbalance at any significance threshold and no intervention.)

2 Supplementary material for Section 4.4

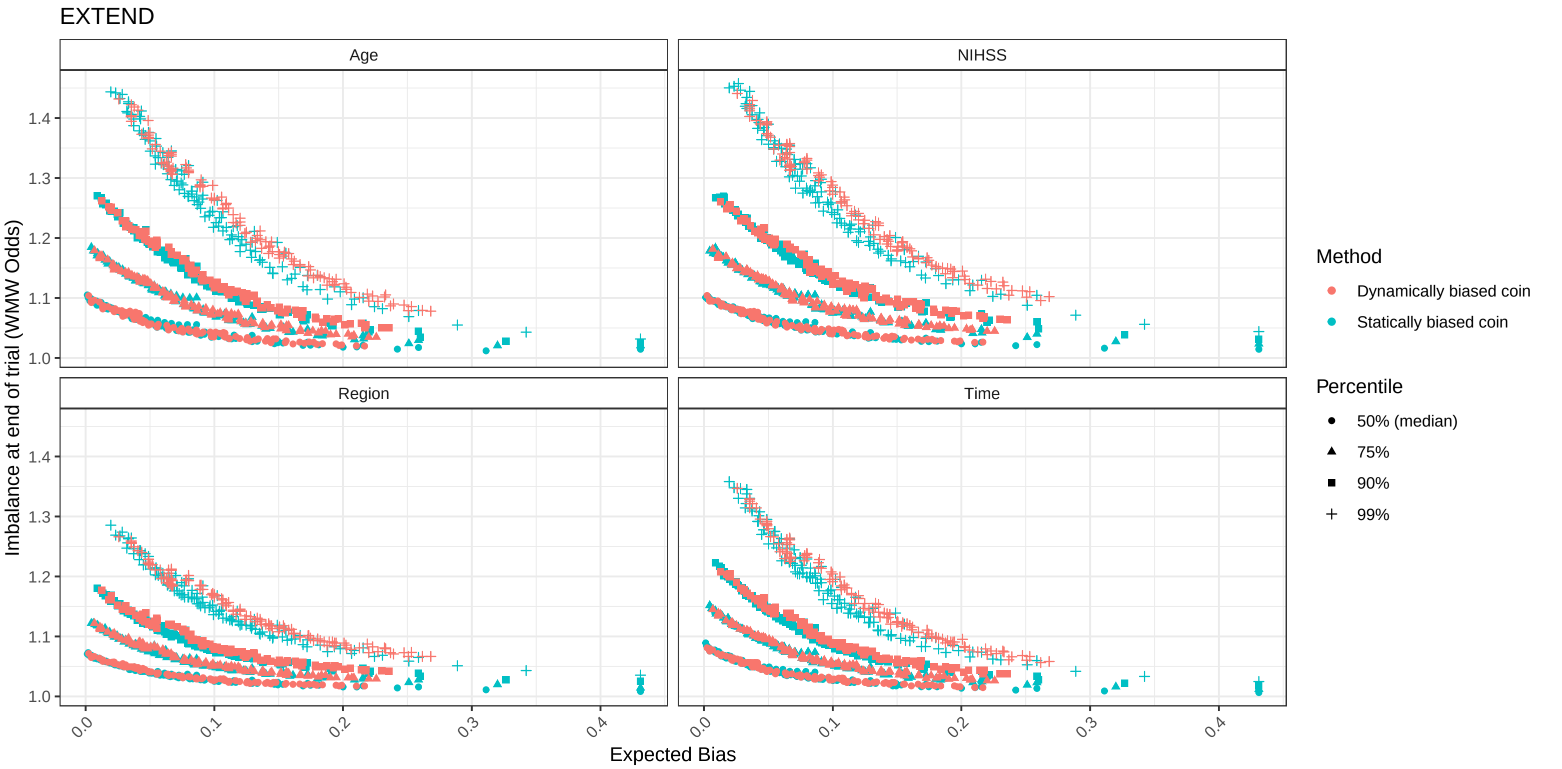

Figure 9: Trade-off curve between covariate imbalance in the EXTEND dataset. Imbalance is measured using WMW Odds. Curves shown give worst-case estimates for this trade-off (i.e. highest percentile imbalance for highest percentile intervention rate. An ideal trial would appear in the lower left-hand corner, with no imbalance and no intervention.)

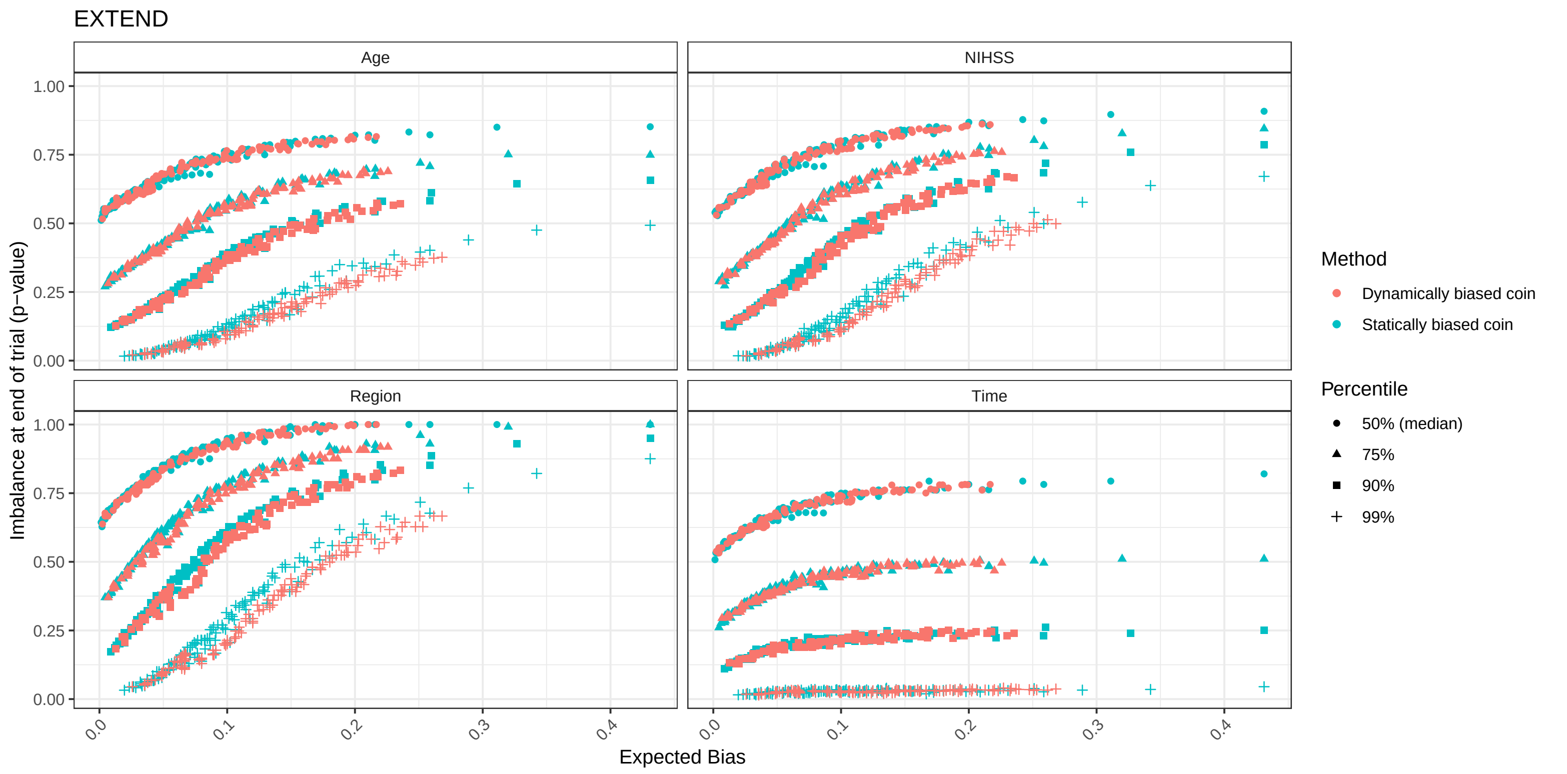

Figure 10: Trade-off curve between covariate imbalance in the EXTEND dataset. Imbalance is measured using conventional statistical tests and reported using p-values. Curves shown give worst-case estimates for this trade-off (i.e. lowest percentile p-value for highest percentile intervention rate. An ideal trial would appear in the upper left-hand corner, with no statistically significant imbalance at any significance threshold and no intervention.)

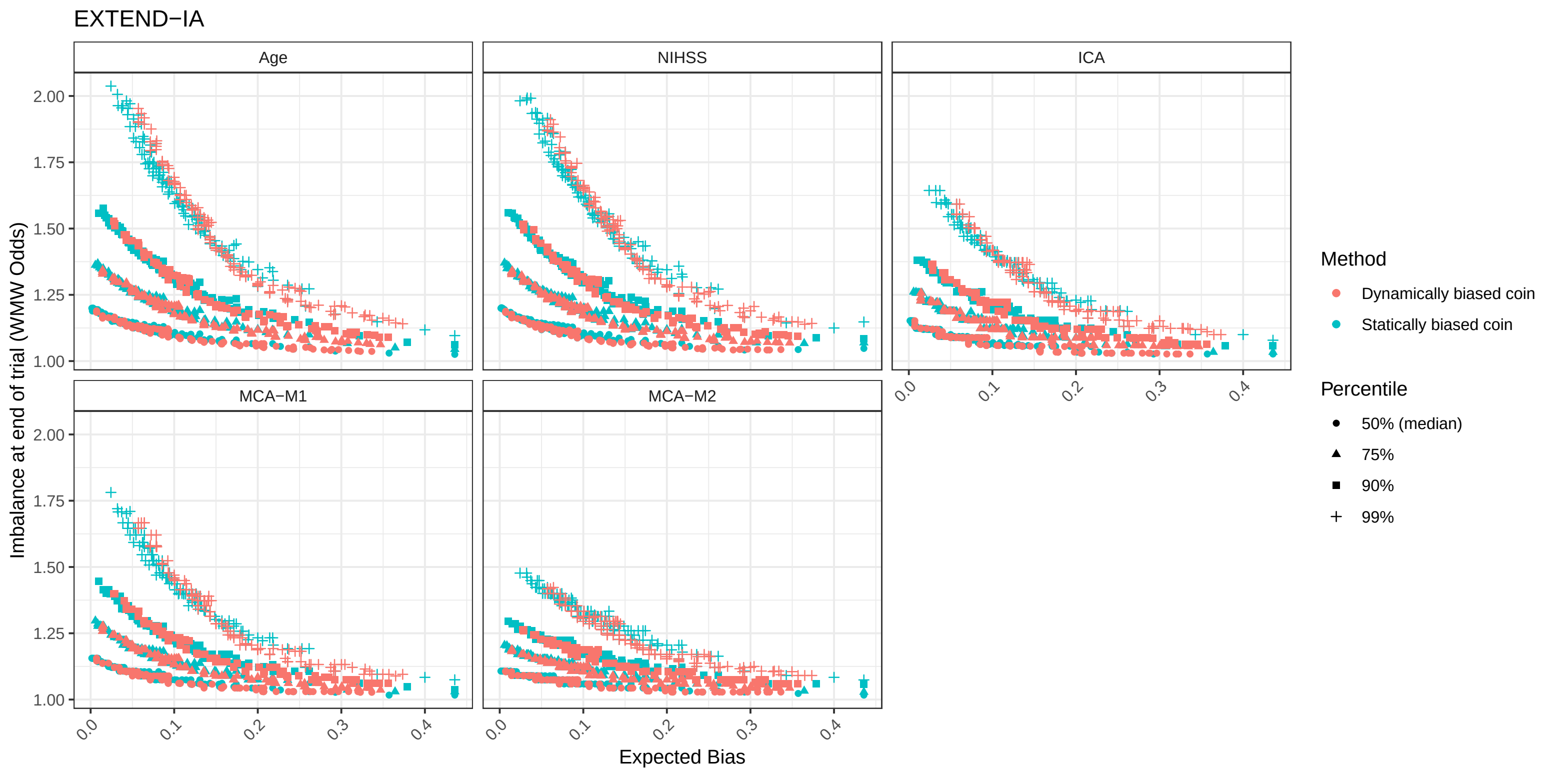

Figure 11: Trade-off curve between covariate imbalance in the EXTEND-IA dataset. Imbalance is measured using WMW Odds. Curves shown give worst-case estimates for this trade-off (i.e. highest percentile imbalance for highest percentile intervention rate. An ideal trial would appear in the lower left-hand corner, with no imbalance and no intervention.)

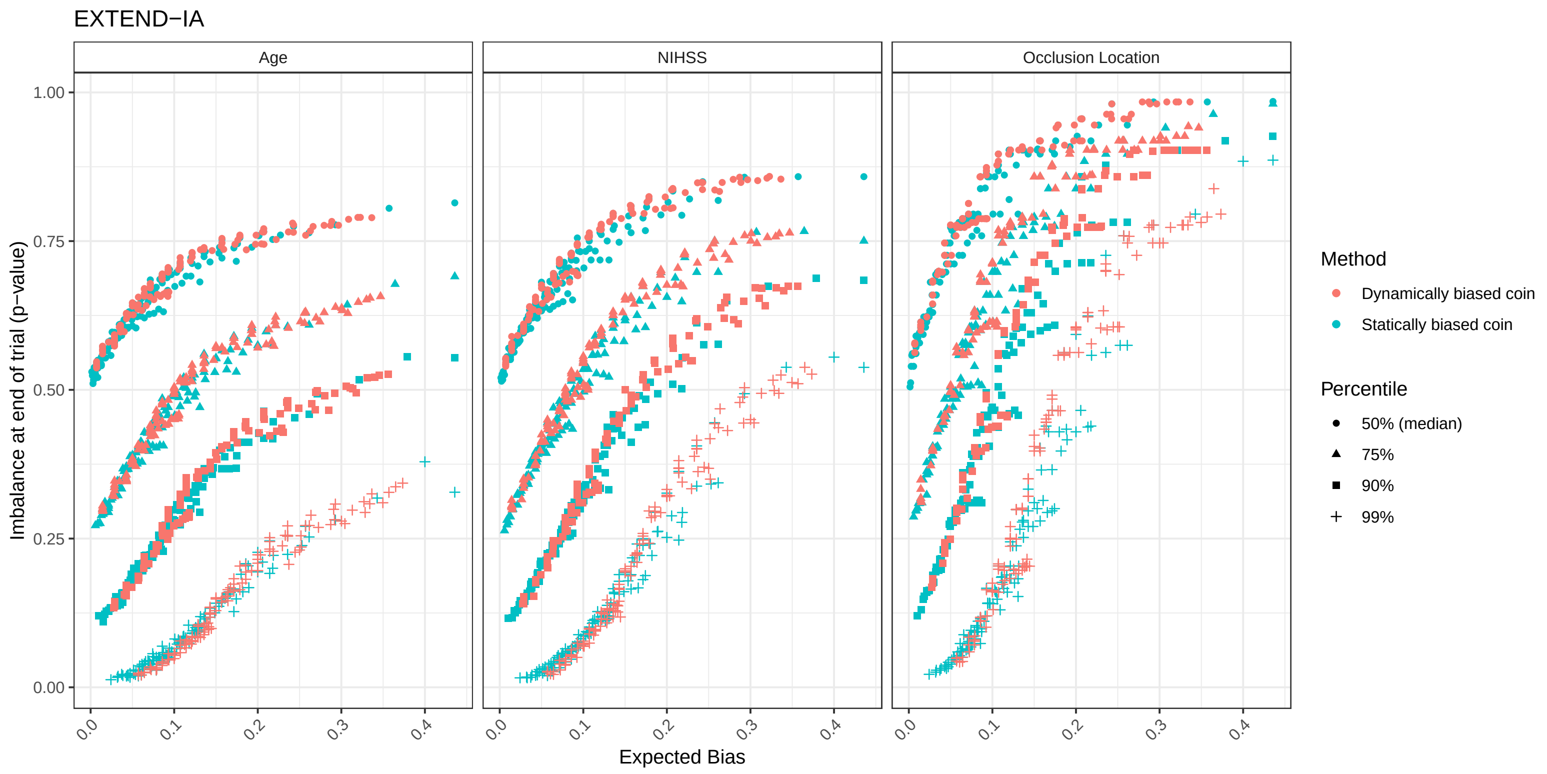

Figure 12: Trade-off curve between covariate imbalance in the EXTEND-IA dataset. Imbalance is measured using conventional statistical tests and reported using p-values. Curves shown give worst-case estimates for this trade-off (i.e. lowest percentile p-value for highest percentile intervention rate. An ideal trial would appear in the upper left-hand corner, with no statistically significant imbalance at any significance threshold and no intervention.)

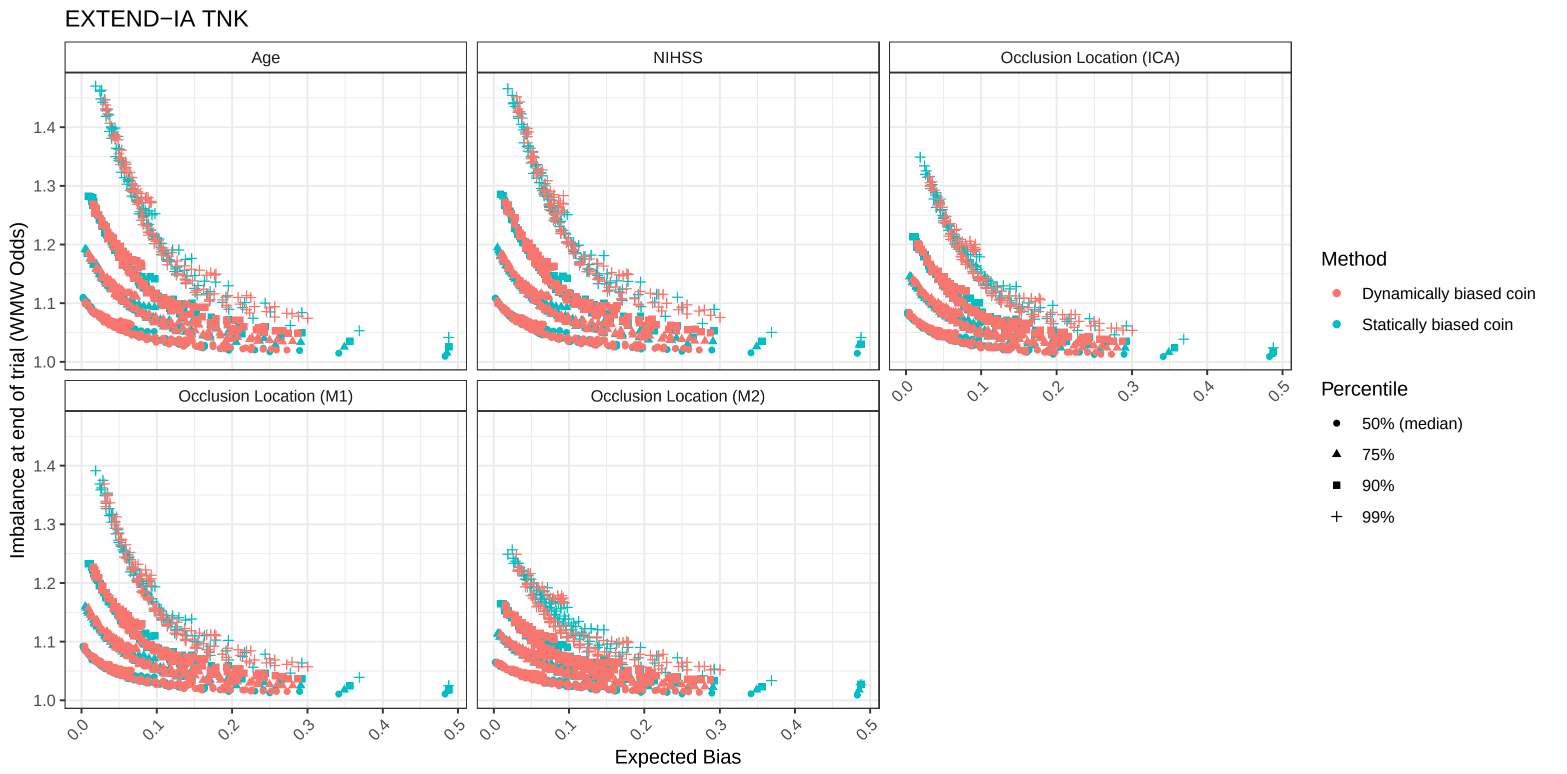

Figure 13: Trade-off curve between covariate imbalance in the TNK dataset. Imbalance is measured using WMW Odds. Curves shown give worst-case estimates for this trade-off (i.e. highest percentile imbalance for highest percentile intervention rate. An ideal trial would appear in the lower left-hand corner, with no imbalance and no intervention.)

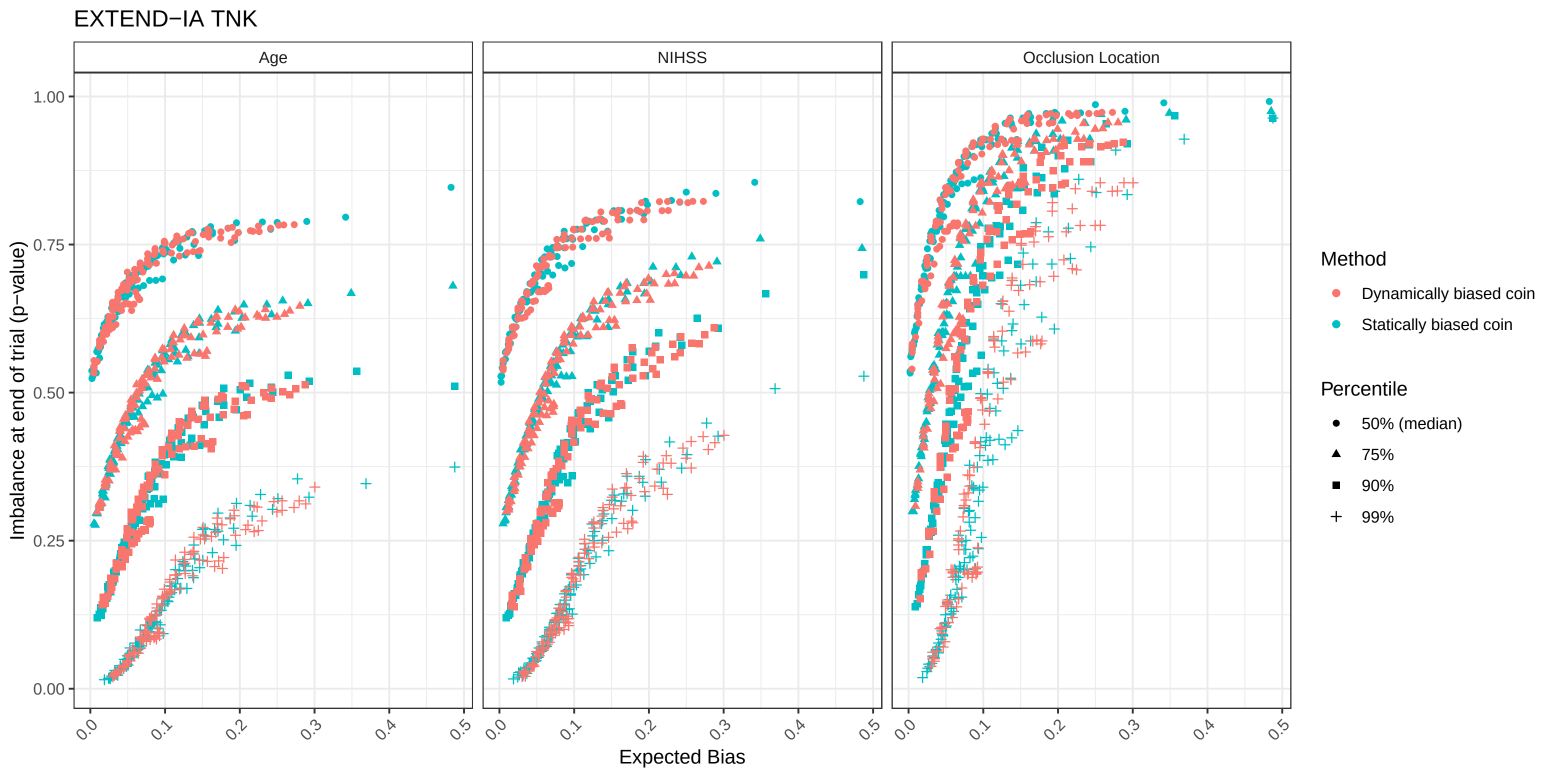

Figure 14: Trade-off curve between covariate imbalance in the TNK dataset. Imbalance is measured using conventional statistical tests and reported using p-values. Curves shown give worst-case estimates for this trade-off (i.e. lowest percentile p-value for highest percentile intervention rate. An ideal trial would appear in the upper left-hand corner, with no statistically significant imbalance at any significance threshold and no intervention.)

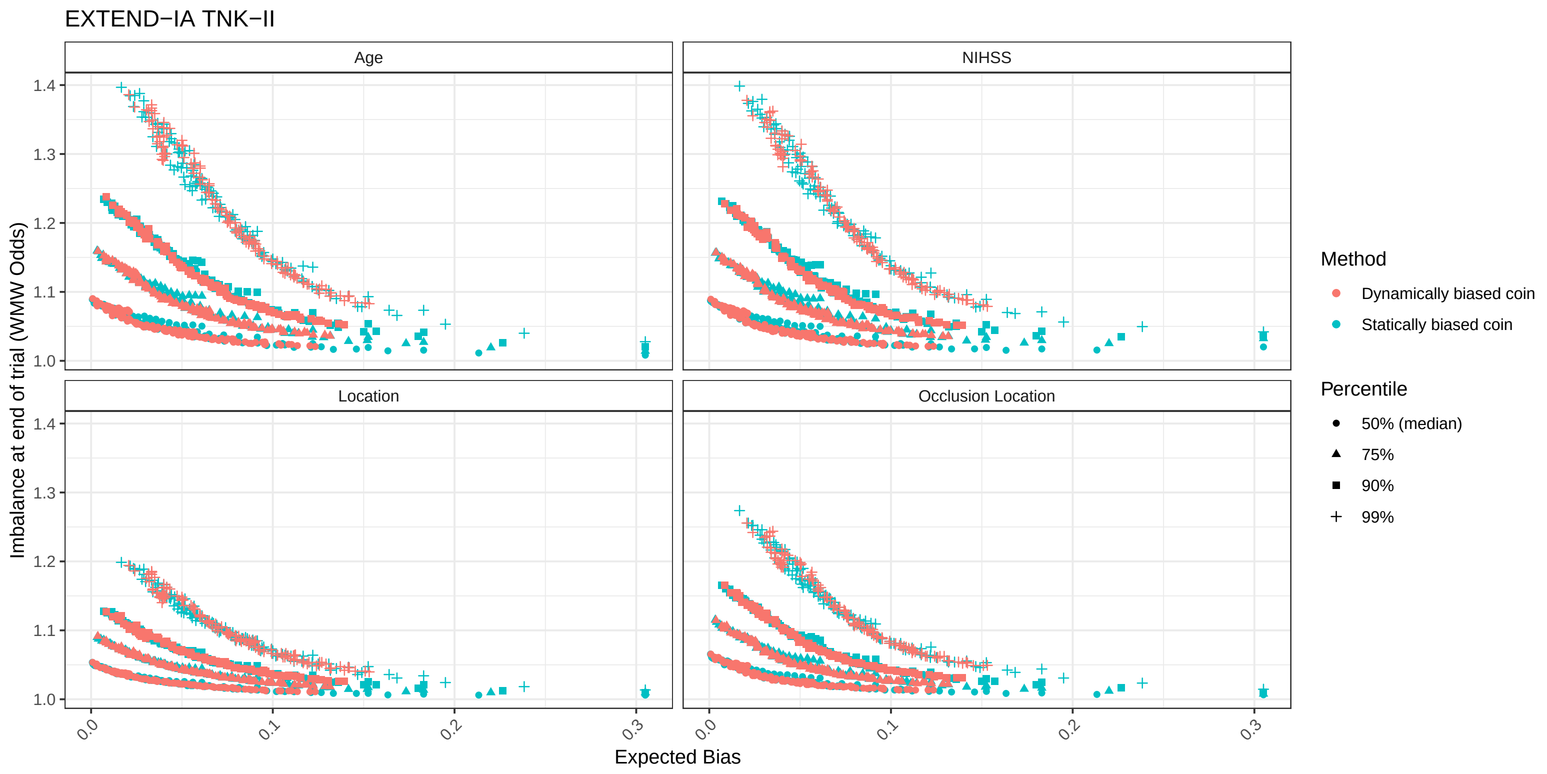

Figure 15: Trade-off curve between covariate imbalance in the TNK Part 2 dataset. Imbalance is measured using WMW Odds. Curves shown give worst-case estimates for this trade-off (i.e. highest percentile imbalance for highest percentile intervention rate. An ideal trial would appear in the lower left-hand corner, with no imbalance and no intervention.)

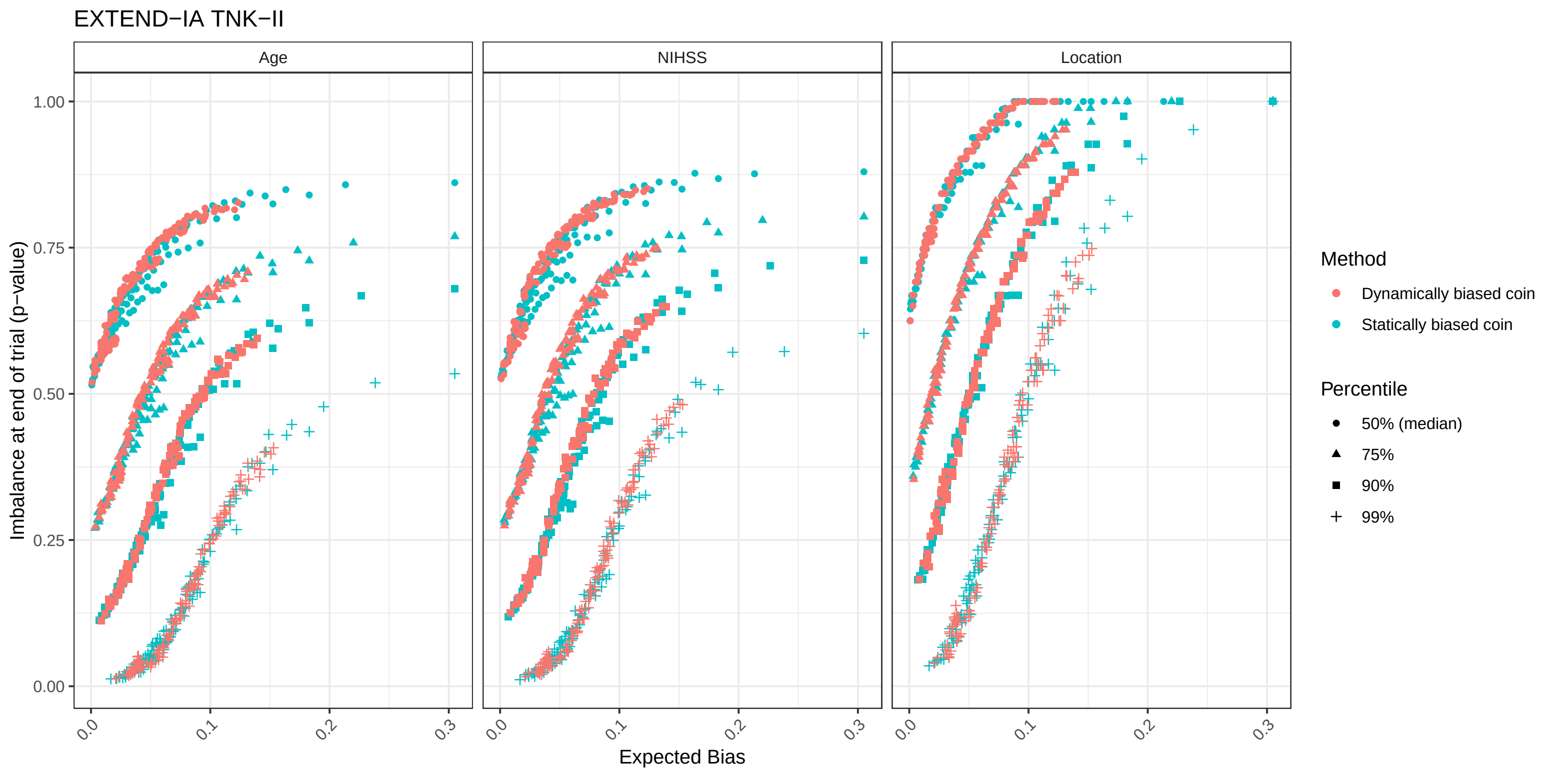

Figure 16: Trade-off curve between covariate imbalance in the TNK Part 2 dataset. Imbalance is measured using conventional statistical tests and reported using p-values. Curves shown give worst-case estimates for this trade-off (i.e. lowest percentile p-value for highest percentile intervention rate. An ideal trial would appear in the upper left-hand corner, with no statistically significant imbalance at any significance threshold and no intervention.)

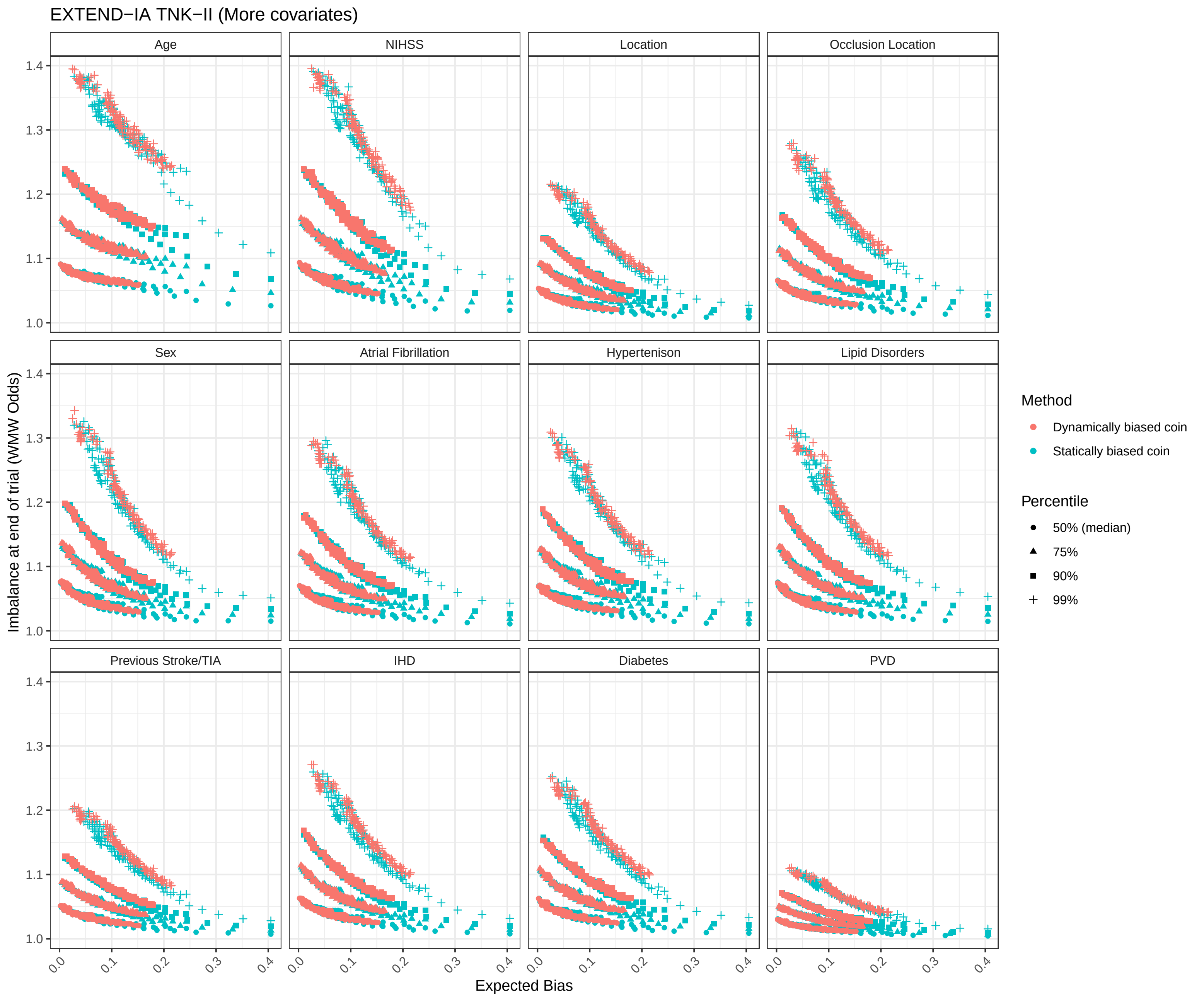

Figure 17: Trade-off curve between covariate imbalance in the expanded TNK Part 2 dataset. Imbalance is measured using WMW Odds. Curves shown give worst-case estimates for this trade-off (i.e. highest percentile imbalance for highest percentile intervention rate. An ideal trial would appear in the lower left-hand corner, with no imbalance and no intervention.)

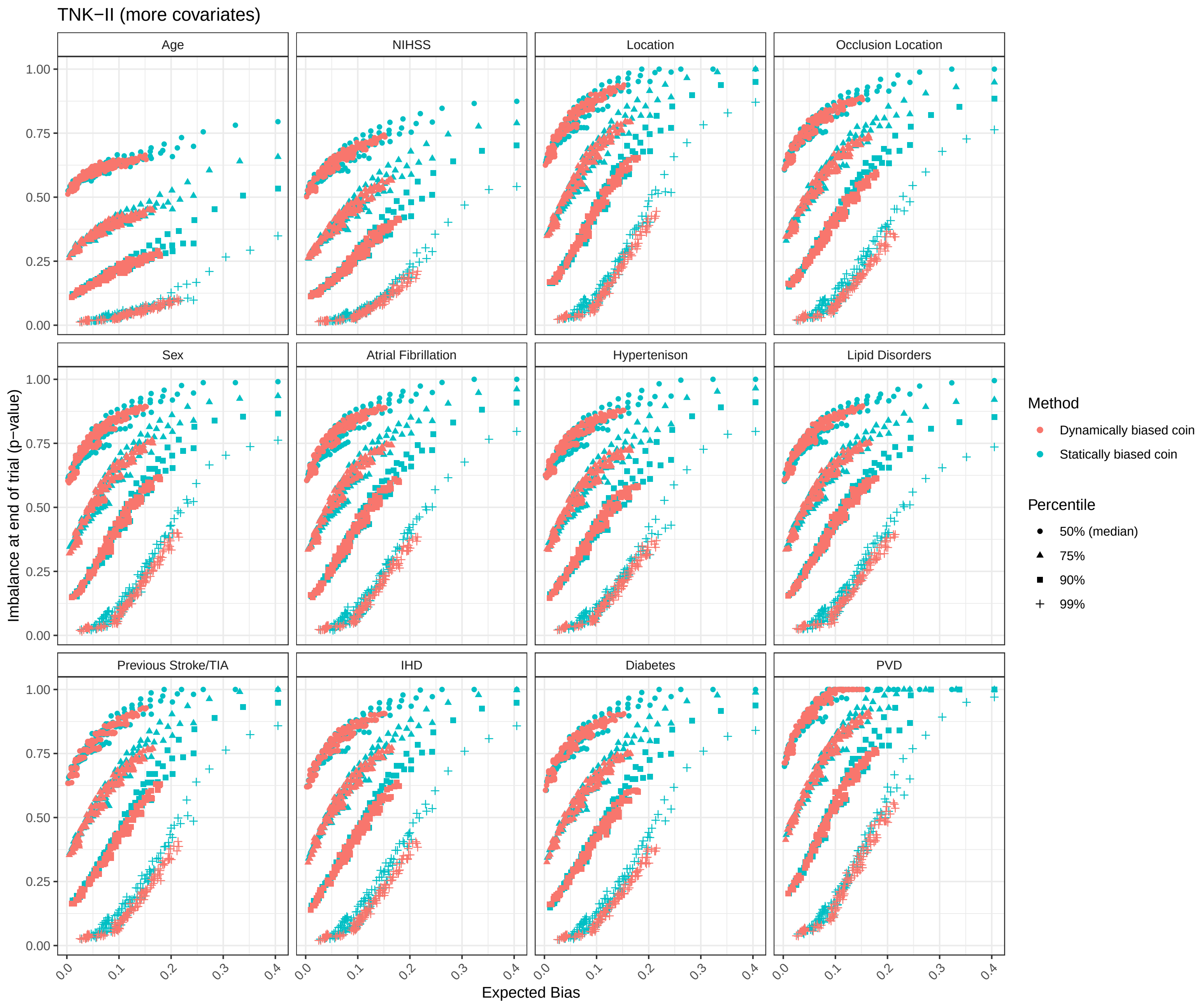

Figure 18: Trade-off curve between covariate imbalance in the expanded TNK Part 2 dataset. Imbalance is measured using conventional statistical tests and reported using p-values. Curves shown give worst-case estimates for this trade-off (i.e. lowest percentile p-value for highest percentile intervention rate. An ideal trial would appear in the upper left-hand corner, with no statistically significant imbalance at any significance threshold and no intervention.)
